# Aging mechanisms—A perspective mostly from *Drosophila*

**DOI:** 10.1002/ggn2.10026

**Published:** 2020-08-10

**Authors:** Amy Tsurumi, Willis X. Li

**Affiliations:** ^1^ Department of Surgery Massachusetts General Hospital, and Harvard Medical School Boston Massachusetts USA; ^2^ Department of Microbiology and Immunology Harvard Medical School Boston Massachusetts USA; ^3^ Shriners Hospitals for Children‐Boston® Boston Massachusetts USA; ^4^ Department of Medicine University of California at San Diego La Jolla California USA

**Keywords:** ageing, aging, aging‐related diseases, *Drosophila*, epigenetics, geriatrics

## Abstract

A mechanistic understanding of the natural aging process, which is distinct from aging‐related disease mechanisms, is essential for developing interventions to extend lifespan or healthspan. Here, we discuss current trends in aging research and address conceptual and experimental challenges in the field. We examine various molecular markers implicated in aging with an emphasis on the role of heterochromatin and epigenetic changes. Studies in model organisms have been advantageous in elucidating conserved genetic and epigenetic mechanisms and assessing interventions that affect aging. We highlight the use of *Drosophila*, which allows controlled studies for evaluating genetic and environmental contributors to aging conveniently. Finally, we propose the use of novel methodologies and future strategies using *Drosophila* in aging research.

## Graphical abstract

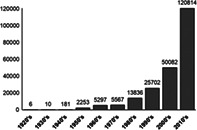

## 1 TOWARD RIGOROUS DEFINITIONS OF THE TERMS USED IN AGING RESEARCH

**FIGURE 1:**
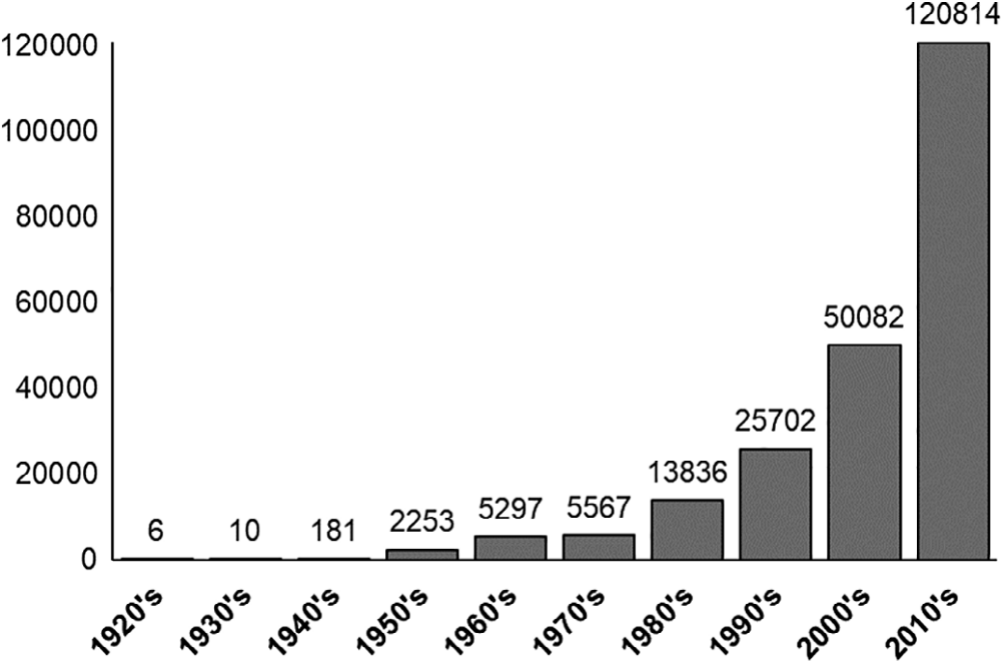
Overall trend in aging‐related publications. The bar graph shows an exponential increase in the articles in the PubMed database with the keywords, “aging/ageing” in the title or abstract (from a search conducted in January 2020)

In the past century, global life expectancy has almost doubled, owing to improved control of infectious diseases and enhanced quality of life.^1^ The United Nations predicts that by 2050, the global geriatric population (age of 80 years old and above) will triple that of 2015.^2^ Given the expected rise in the proportion of the elderly to the young, studies to elucidate mechanisms that promote healthy aging will be of paramount impact. Indeed, there has been a remarkable upsurge in the number of the articles related to aging in the past decade (Figure 1), reflecting intense interest in aging research. However, a plethora of challenges and questions remain to be addressed.

### 1.1 Aging contribution to aging‐related diseases

One of the most challenging questions in aging research is to distinguish the mechanisms and even phenotypes of aging from those of aging‐related diseases. Aging is a risk factor for a multitude of chronic conditions and diseases, including cardiovascular diseases, osteoporosis, dementia, osteoarthritis, type 2 diabetes, cancer, and chronic obstructive pulmonary disease (COPD).^3^ The diversity of these diseases suggests that the cellular and physiological changes that determine the onset of aging‐related diseases are highly complex. While some suggest that aging is a distinct syndrome that should be considered separately from aging‐related diseases, others argue that these two overlap in underlying mechanisms to varying degrees.^2^, ^4^ The lack of consensus may underscore the necessity to characterize the natural aging process.^5^ These aging‐related diseases can also manifest in those who are neither old in age nor physiologically aged. For example, cancers could also occur among young people.^6^ Another example is Alzheimer's disease (AD), in which early‐ and late‐onset are considered to have separate etiologies,^7^ perhaps due to different genetic backgrounds. As such, it is beneficial to have models for evaluating the relative degree of contribution of aging to diseases.

**FIGURE 2:**
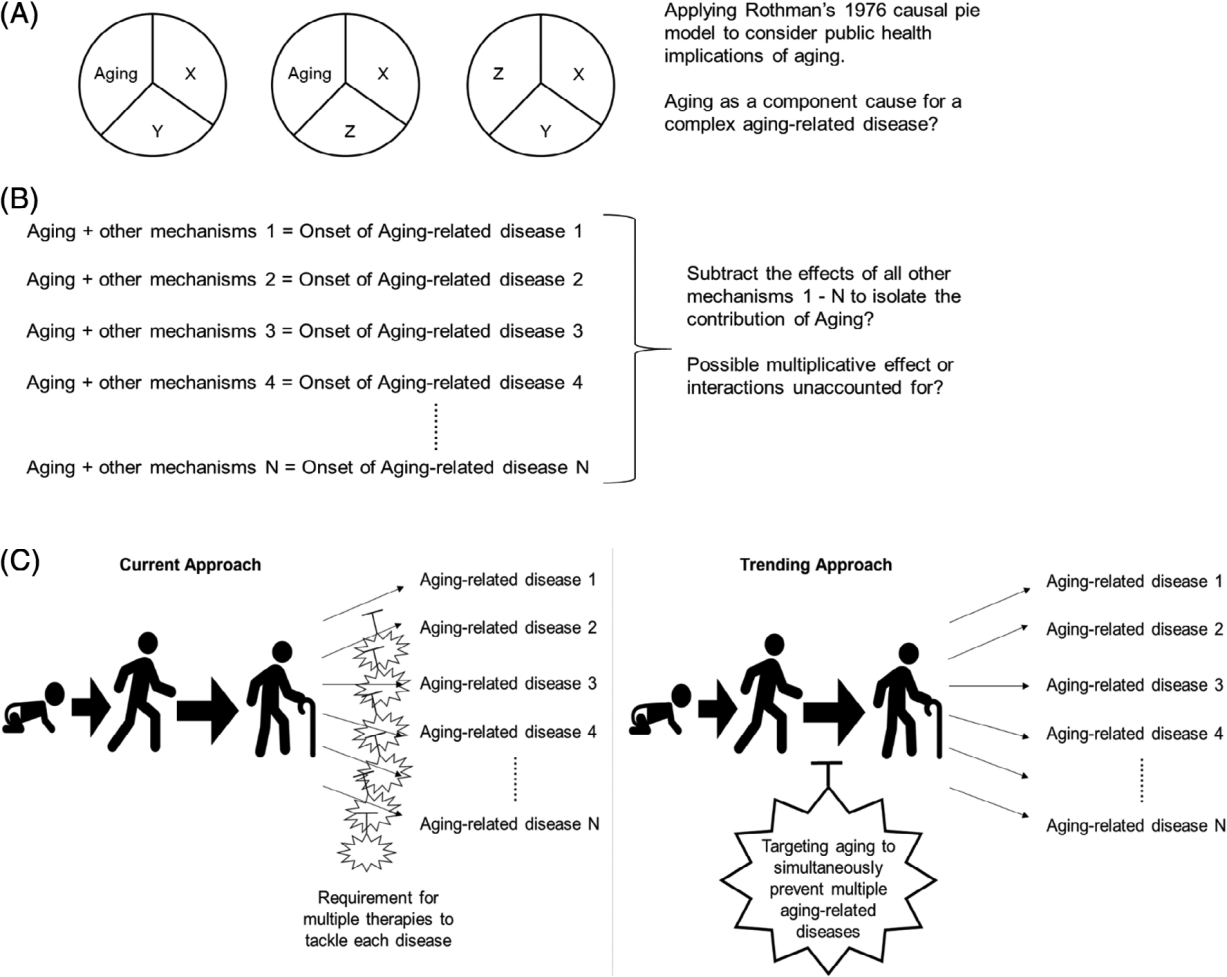
Possible models to evaluate the contribution of the molecular consequences of aging to the onset of aging‐related diseases. A, Applying Rothman's 1976 causal pie model^8^ to understanding the mechanisms of the onset of aging‐related diseases and the public health implications of targeting aging. B, Possible strategy for isolating the effect of aging on the outcome of various aging‐related diseases. It may also be important to elucidate possible multiplicative effects or interactions of aging with other variables, in order to address the degree of contribution of aging to aging‐related diseases. C, The current vs trending approach to tackle aging‐related diseases. A trending approach focuses on preventing aging‐related diseases by targeting aging itself, rather than therapeutic interventions directed at one specific aging‐related disease at a time

Rothman's 1976 causal pie model^8^ was initially employed to model causal inference in the field of epidemiology. We propose that this method can also be applied to describe the causal relationship between aging and aging‐related diseases (Figure 2A), and this application may aid scientists in describing and quantifying the public health relevance to aging. We suggest considering aging or the cellular and physiological consequence of aging as a “component cause” (ie, a slice of a pie representing a factor that is not sufficient on its own to lead to the disease onset) of an aging‐related disease, which explains an increased incidence among older individuals. However, since the disease can also occur in young people, in this model, aging on its own is not considered as a “necessary cause” (ie, a slice present in all the pies, representing a factor that must be present for the disease onset). The “sufficient causes” (ie, complete pies that represent combinations of all the factors that contribute to the disease onset) thus represent different pathways toward the disease onset. The impact of targeting aging on public health can be conceptualized by reducing the proportion of “sufficient causes.” Methods have been developed to apply epidemiological data to causal pie models to quantify the burden of different classes of “sufficient causes” and the effect of interventions on a given “component cause,”^9^, ^10^, ^11^ which may also be used for the aging field.

Considering the diversity in aging‐related diseases, finding shared molecular mechanisms may aid in isolating the effect of aging (Figure 2B). Such approach is not trivial, as the effect of aging may be additive, or multiplicative, depending on other variables. Previous therapeutic strategies focus on one aging‐related disease at a time and after its onset. In contrast, targeting aging can prevent or delay the onset of an array of aging‐related diseases simultaneously, therefore becoming an important alternative (Figure 2C). Studies using model organisms such as *Drosophila* can help provide mechanistic insights in the aging process and its interaction with diseases.

### 1.2 Lifespan and healthspan as distinct aging phenotypes

While “lifespan” refers to the time from birth to death, “healthspan” refers to the length of healthy life. Current goals for aging research emphasize on elucidating the interventions that delay the onset of diseases to extend healthspan.^1^, ^5^ However, the extension in healthspan is only meaningful when being related to the lifespan. Recently, sub‐definitions of healthspan extension—“chronological” vs “proportional”—were coined.^12^ “Chronological” healthspan extension describes a delayed onset of morbidity proportionally with a prolonged lifespan. Therefore, the total morbidity time is shortened. “Proportional” healthspan extension refers to the total morbidity time that remains the same despite a delay in the onset due to extended lifespan.

In human cohort studies, it is challenging to quantify the healthspan extension due to the difficulty in obtaining health records for all different competing morbidities, as well as a lack of a clear consensus of the conditions that constitute morbidity and warrant intervention. For example, geriatric patients often experience circadian rhythm shift,^13^, ^14^ however, this symptom is often unreported or not considered as a significant morbidity to be included in measuring healthspan. Thus, a recent publication^5^ demonstrated a table that indexed healthspan measures based on the World Health Organization's codified patient classification systems, various physiological measurements, other reported measures of frailty such as the Healthy Aging Index,^15^ Successful Aging Index,^16^ and Healthy Aging Score.^17^ It also summarizes the evidence found in model organisms such as mice and *Caenorhabditis elegans*. Below, we propose to extend this chart by including relevant *Drosophila* phenotypes and the assays measuring them (part 2b).

## 2 ASSAYS AND METHODOLOGIES FOR THE AGING RESEARCH IN *DROSOPHILA*

### 2.1 *Drosophila* as a model organism for aging studies

The trend using model organisms in aging research have shifted from ascertaining only longevity (lifespan) to also include physiological assessments (healthspan).^18^ *Drosophila melanogaster* is a genetically tractable model organism with a long history in aging research. The first documented aging experiment was conducted in 1916, which assessed the effect of temperature and food composition.^19^ Using *Drosophila*, the pro‐ or anti‐aging effects of environmental factors and pharmacological interventions can be easily assessed. Since a large number of flies with the same genotype can be investigated under controlled conditions, it allows scientists to conduct statistically powerful experiments at low cost. The fly model is also amenable to largescale screens for new interventions and test the combinations of interventions quickly. Human cohort studies of environmental factors are notoriously susceptible to confounding and reverse causation. The *Drosophila* model system can help validate the findings in human studies and establish causality. The short lifespan is another major advantage. The typical lifespan of *Drosophila* in the optimistic temperature of 25°C is approximately 60 days on average. Increasing the temperature to 29°C leads to a reduced lifespan, while decreasing it to 18°C does the opposite.^20^

Moreover, *Drosophila* is a convenient genetic system with numerous mutants and transgenic tools. For example, the well‐established UAS/GAL4 system allows tissue‐specific transgene ectopic expression^21^ and temporal control can also be achieved using a temperature or hormone‐inducible variant of the system.^22^, ^23^ RNAi knockdown studies can be conducted easily using a curated collection of RNAi lines that target most transcripts.^24^ In addition, the CRISPR‐Cas9 tools for gene editing in vivo have been developed in *Drosophila*.^25^ Using these methods, scientists have effectively conducted the mechanistic studies, which are challenging in humans, to understand conserved genetic pathways implicated in human aging. Such pathways include sirtuins and other chromatin regulators (Table 2), and those involved in regulating inflammation,^26^ oxidative stress detoxification,^27^ insulin signaling,^28^ and mammalian Target of Rapamycin (mTOR) pathway.^29^

### 2.2 Applying fly‐specific assays to the understanding of human healthspan index

Various assays have been developed in *Drosophila* for observing aging phenotypes and aging‐related disease models, which may relate to human healthspan (^5^; Table 1). For example, survival curve measurements are standard methods for assessing lifespan. The assessment of aging‐related functional decline or functional senescence aids the quantification of the healthspan extension resulting from specific interventions.^30^ Investigators may combine both measurements to evaluate how an intervention affects lifespan and healthspan.

**TABLE 1:** Suggested assays in *Drosophila* to investigate traits that contribute to healthspan in humans

Healthspan traits conserved between *Drosophila* and humans	*Drosophila* phenotype concept in the Gene Ontology (GO)	Relevant assays to investigate healthspan traits in *Drosophila*
*Physiological function*		
Stress resistance	GO:0006950—response to stress	Survival kinetics in the presence of environmental stressors
Thermotolerance heat shock tolerance	GO:0009408—response to heat	Heat shock survival kinetics^41^
Hypoxic stress tolerance	GO:0001666—response to hypoxia	Survival in hypoxic environment.^54^
Oxidative stress tolerance	GO:0006979—response to oxidative stress	Survival kinetics fed with paraquat^55^ and other oxidative stress‐inducers
Metabolic status Metabolic homeostasis Metabolic pathology	GO:0019222—regulation of metabolic process	Assays to quantify metabolites, mitochondrial activity, and other markers of metabolic state^273^, ^274^, ^275^
Redox status Redox homeostasis Redox pathology	GO:0051775—response to redox state	Assays to quantify reactive oxygen species^276^, ^277^
Immune status Immune homeostasis Immune pathology	GO:0006955—immune response	Assays to quantify innate immunity and phagocytic response
*Both physical strength and cognitive function*		
Motivated locomotion Stimulated locomotion	GO:0007626—locomotion in response to stimulus	Geotaxis assay^31^, ^32^, ^33^ with shaking Response to light, olfaction, and taste^44^ Variations of olfactory maze tests^278^, ^279^, ^280^ Taste preference assay^281^ Real‐time imaging of response to gentle pricking and rotation, odor‐stimulated movement, and neuroactivity imaging^282^
Motor balance dexterity	GO:0050885—regulation of balance	High‐speed real‐time machine vision robot developed for *Drosophila* to capture behavioral response to gentle pricking and rotation, odor‐stimulated movement, and neuroactivity imaging^282^
Cellular integrity: Muscle Neuronal Intestinal Pathological loss of cell integrity	GO:0046716—muscle fiber maintenance GO:0070050—neuron maintenance GO:0060730—epithelial structure maintenance	Muscle fiber integrity by actin filament staining^32^ Brain degeneration vacuoles^31^, ^41^, ^42^ Retinal degeneration and loss of ommatidium photoreceptor integrity^41^, ^42^ Neuromuscular junction (NMJ) marker abnormality quantification^42^, ^43^
*Physical strength*		
Locomotion Unmotivated Unstimulated	GO:0040011—locomotion (self‐propelled)	Geotaxis assay^31^, ^32^, ^33^ with no shaking Variety of flight duration^33^, ^34^ and exploratory activity tests^33^, ^35^, ^36^
Grip strength		Measured in mice, but not a conserved trait in *Drosophila*
Gait speed, chair rising		Similar traits measured in mice, but not a conserved trait in *Drosophila*
Muscle integrity pathological loss of muscle integrity	GO:0046716—muscle fiber maintenance	Muscle fiber integrity by actin filament staining^32^
*Cognitive function*		
Sensory perception	GO:0007600—sensory perception	Response to light, olfaction and taste^44^ Variations of olfactory maze tests^278^, ^279^, ^280^ Taste preference assay^281^ Odor‐stimulated movement real‐time imaging^282^
Short‐term memory processing speed	GO:0007614—short‐term memory	Response to light, olfaction, and taste^44^ Variations of olfactory maze tests^277^, ^278^, ^279^ Various olfactory and taste memory tests^45^, ^46^, ^47^
Sleep defective	GO:0030431—sleep	*Drosophila* Activity Monitor (DAM) device and accompanying analysis software called ShinyR‐DAM^283^ Locomotor activity monitoring and phase‐response curves^284^ Sleep behavior assay^285^
Cardiac rhythm	GO:0042749—regulation of circadian sleep/wake cycle	Heart rate measurement^52^
Executive function verbal function		*Drosophila* is not verbal
Neuronal integrity neuronal pathology	GO:0070050—neuron maintenance	Brain degeneration vacuoles^31^, ^41^, ^42^ Retinal degeneration and loss of ommatidium photoreceptor integrity^41^, ^42^ Neuromuscular junction (NMJ) marker abnormality quantification^42^, ^43^ Neuroactivity real‐time imaging^282^
*Reproductive function*		
Number of offspring Offspring health Surviving offspring	GO:0019953—sexual reproduction	Eggs laid, number of progeny relative to copulation^48^, ^49^ Quantify germ line stem cell number to evaluate reproductive function^50^, ^51^

*Note*: These assays can be used to assess the effects of genetic mutations, epigenomic alterations, and environmental conditions to understand the basic biological mechanisms of aging in *Drosophila*. (Adapted from Table 2 in Reference 5 under CC‐BY 4.0 attribution license).

Among commonly used assays for assessing healthspan are those that measure movement. These include the negative geotaxis assay for evaluating climbing ability,^31^, ^32^, ^33^ and a variety of flight duration,^33^, ^34^ and exploratory activity tests.^33^, ^35^, ^36^ The movement weakening during aging may be due to skeletal muscle deterioration or cognitive decline and can be further examined using histological methods. For example, staining of actin filaments help visualize the loss of muscle fiber integrity,^32^ and various neuronal markers allow the detection of the loss of specific neurons.^37^, ^38^, ^39^, ^40^ Other aging‐related neurological phenotypes can be also quantified, including brain degeneration vacuoles,^31^, ^41^, ^42^ retinal degeneration and loss of ommatidium photoreceptor integrity,^41^, ^42^ and neuromuscular junction abnormalities.^42^, ^43^ Aging‐related cognitive decline can be assessed by evaluating the fly response to light, olfaction and taste,^44^ and memory and learning.^45^, ^46^, ^47^

Reproductive decline also occurs during aging and can easily be investigated by quantifying egg‐laying or the number of progeny relative to copula copulation.^48^, ^49^ Additionally, germ line stem cells can be stained and quantified.^50^, ^51^ Cardiac function assays include monitoring heart rate^52^ and cardiac tube wall movements.^53^ Heat shock survival kinetics^41^ or survival in hypoxic environment^54^ can be used to assess the resistance to stress. Another commonly used assay is to evaluate oxidative stress resistance through the survival on paraquat.^55^ Assays were also developed for assessing the impact of social interaction on lifespan.^56^

### 2.3 Using *Drosophila* for the mechanistic studies of gene‐environment interactions

A large number of aging studies have been conducted in *Drosophila* to characterize the effect of exposure to various environmental agents (notably, diet, microbial interactions, pharmacological interventions, and pollutants) on aging. Such strength is best exemplified by the studies investigating the aging impact of dietary restriction (DR), pharmacological and genetic manipulation of *Sirtuin* family of histone deacetylases (HDACs), mammalian target of rapamycin (mTOR) signaling, and insulin signaling. Here we highlight the aging studies in *Drosophila* that helped the mechanistic understanding in mammals.

#### 2.3.1 Dietary Restriction

It is well‐established that DR enhances lifespan, and this appears to be conserved across species ranging from yeast to invertebrates to rodents. However, whether this conservation is simply due to reduced caloric intake remains elusive. Several *Drosophila* studies have shown that the effect was determined by the relative composition of yeast, sugar and essential amino acids (EAAs), rather than a mere reduction in calories.^57^, ^58^, ^59^, ^60^, ^61^ One study found that supplementing with EAA negated the lifespan extending effect of DR, thus isolating the relevant dietary component,^60^ and interestingly, this effect was accompanied by reduced fecundity.^60^ Another study found that a short‐term DR was also effective, suggesting that the intervention later in life in humans might be a valuable method.^62^ Recently, a randomized control trial of DR, the Comprehensive Assessment of Long‐term Effects of Reducing Intake of Energy (CALERIE) study was conducted, in which healthy and young subjects were randomly assigned to 2 years of caloric restriction or control diet.^63^, ^64^, ^65^, ^66^, ^67^, ^68^, ^69^, ^70^ Ongoing analyses of physiological measurements and molecular profiling of biobank tissue sample are expected to yield invaluable information about the effect of DR on humans. Nevertheless, since controlled experiments and isogenic models are not feasible in human studies, using *Drosophila* is still expected to be advantageous for elucidating mechanistic understanding.

#### 2.3.2 Sirtuins and Dietary Restriction

The link between aging and the *sirtuin* family of histone deacetylases (HDACs) have been controversial. Studies in *Drosophila* helped draw the conclusion that the expression level of *Sirtuin 2* (*Sirt2*) is important for the effect (described in detail in Section 3.1). For instance, the lifespan extension by DR was abrogated in *Sirt2* mutant flies, and different dietary conditions altered the magnitude of the effect.^71^ Such findings again highlight the advantage of using the *Drosophila* model for meticulous and controlled studies of gene‐environment interactions, which are challenging in humans. Additionally, the *Drosophila* model was advantageous in ascertaining the lifespan extension effects of the *Sirtuin*‐activating compounds including resveratrol, fisetin, and alkylresorcinols.^72^, ^73^

#### 2.3.3 mTOR signaling and dietary restriction

*Drosophila* has proven to be advantageous for understanding the impact of the mTOR signaling pathway on aging genetically, pharmacologically, and under different dietary conditions. It is well‐established that mTOR is a master regulator of protein synthesis and cell growth, and that an augmented mTOR signaling is negatively correlated with lifespan in multiple species.^74^ It has been demonstrated that supplementing with EAA negates the lifespan extending effect of DR by upregulating mTOR, while rapamycin, a mTOR inhibitor abolished this effect and extend the lifespan.^60^ Further characterization of tissue‐specific gene expression changes identified GATA transcription factors, the zinc finger DNA binding proteins, as the downstream mediator of mTOR signaling^59^; GATA transcription factors have been shown to regulate lifespan in multiple organisms. These studies exemplify the strength of combining genetic tools with pharmacological treatments to dissect molecular mechanism of aging.

#### 2.3.4 Insulin/IGF and JNK/foxo signaling, metabolism, and dietary composition

It is well‐established that reducing insulin/insulin‐like growth factor (IGF) signaling extends lifespan across species, including humans and *Drosophila*, and that one major signaling pathway mediating the effect is the c‐Jun N‐terminal Kinase (JNK)/*Forkhead box and sub‐group O* (*foxo*) pathway.^75^, ^76^, ^77^ The JNK/foxo pathway is a conserved mechanism that confers protection against stress, as well as counters the activity of Insulin/IGF signaling.^28^ Consistent with this notion, a study found that in the fly head, the number of genes regulated by the Foxo transcription factor significantly decreased with age, and many Foxo‐targeted genes were also altered in their expression profile (up‐ or downregulation) during aging.^78^ Moreover, a high sugar diet that reduces lifespan was found to augment the insulin/IGF signaling and reduce the Foxo signaling.^79^

Studies using the *Drosophila* model organism underscore the importance of considering various environmental exposures that interact with this pathway. For instance, while overexpressing *foxo* in the head's fat body leads to lifespan extension, it was found that this only occurred under a high level of dietary yeast.^80^ Moreover, this study showed that *foxo* heteroallelic null mutants on DR had the lifespan comparable to wildtype controls, suggesting that the lifespan extension effect of DR may not be mediated by Foxo.^80^ DR or JNK/Foxo signaling could also regulate separate pathways to extend lifespan; DR was found to reduce the expression of insulin‐like peptide‐5 (ilp5), while *foxo* overexpression lead to the reduction of insulin‐like peptide‐2 (ilp2). Another study showed that different food concentrations are required for wildtype and the insulin receptor substrate, *chico* mutants to achieve a maximum median of lifespan.^81^ This study suggests that the genetic effects of longevity may depend on food composition.

Another gene that was found to prolong lifespan in *Drosophila* is *I*'*m not dead yet* (*Indy*). *Indy* encodes a plasma membrane transporter of Krebs cycle metabolites.^82^ Given the well‐established role of metabolism in aging across species, this finding may be relevant widely. By simultaneously assessing whether there is a trade‐off between lifespan extension and healthspan measures of fecundity, locomotion and metabolism, the study found that increased lifespan with *Indy* mutation did not alter healthspan measures under normal food conditions, however, the reduced fecundity occurred under the DR conditions. This suggests that the trade‐off between reproduction and lifespan depend on both environmental conditions and genetic susceptibility. Together, these studies highlight the advantage of using *Drosophila* to gain mechanistic insights in the conditions that affect both lifespan and healthspan.

## 3 EPIGENETIC MECHANISMS IMPLICATED IN *DROSOPHILA* AND HUMAN AGING

The mechanisms involving epigenetic regulation (histone modifications, DNA methylation, noncoding RNAs, RNA modifications) are among the major models of aging, presented as the “hallmarks” of aging^83^ and the “seven pillars” of aging by the NIH steering conference on aging.^84^ The genetic heritability of longevity in humans is estimated to be only 15% to 40%,^85^ suggesting the likely significant contribution of epigenetic and environmental factors. In this section, we present major studies describing different epigenetic mechanisms in aging, particularly those conserved between *Drosophila* and humans. These studies are summarized in Tables 2, 3, 4.

**TABLE 2:** Histone‐related mechanisms conserved between *Drosophila* and human aging

Species	Epigenomic modifications	References
	*Histone methylation—transcriptional repression marks*	
	H3K9me3 colocalized with HP1a in heterochromatin	
*Drosophila*	Changes in genomic distribution of H3K9me3 and HP1 with age High H3K9me3 and HP1a (encoded by *Dme‐Su(var)205*) enrichment in heterochromatin loci (pericentric, fourth chromosome, facultative heterochromatin islands) in young flies, and reduction in aged flies In other regions, the opposite trend was detected	[91]
	Reduced lifespan and healthspan (vertical velocity and muscle integrity) with reduced HP1a due to *Dme‐Su(var)*205 loss‐of‐function; increased lifespan and healthspan with HP1a overexpression Reduced heterochromatin at rRNA locus, leading to increased rRNA transcription, ribosome biogenesis and protein synthesis with aging	[32]
	Tyrosine unphosphorylated Stat92E (encoded by *Dme‐Stat92E*) promoted position effect variegation via heterochromatin formation whereas JAK kinase gain of function in *Dme‐hop* disrupted heterochromatin (AU: can you please rephrase this sentence? ED)	[96]
	*Dme‐Su(var)205 (encoding Hp1a)* or *Dme*‐*Su(var)3‐9* (encoding histone‐lysine *N*‐methyltransferase Su(var)3‐9), RNAi knockdown of either gene resulted in male germ‐line stem cell loss	[51]
	*Dme*‐*Su(var)3‐9* overexpression increased lifespan and repressed transposable element (TE) elevation in the head and fat body seen during aging	[93]
	*Dme*‐*Su(var)3‐9* mutant females have shorter lifespan compared to wildtype control *Dme*‐*Su(var)3‐9* mutants had disrupted heterochromatin and genome instability, displaying increased DNA damage and chromosomal defects	[95]
	A drug screen found methotrexate to promote heterochromatin formation by increasing H3K9me3 and HP1a foci Methotrexate reduced the eye overgrowth phenotype resulting from JAK/STAT signaling elicited by transgenic overexpression of a ligand, *unpaired* (*Dme‐upd1*)	[107, 286]
	*Ras85D* hyperactivation (*Dme‐Ras85D*) with mitochondrial dysfunction could induce senescence, and lead to increased H3K9me3	[123]
Human	Reduced bulk H3K9me3 in hematopoietic stem cells (HSCs) from elderly subject compared to young	[287]
	Loss of function mutation of *Hsa‐LMNA* encoding lamin A/C reduced H3K9me3 and HP1γ *Hsa‐LMNA* mutation causes the Hutchinson‐Gilford progeria syndrome (HGPS, OMIM 176670)	[288]
	Restoring *Hsa‐LMNA* splicing restores nuclear lamina defect and normal levels of H3K9me3 and HP1γ (encoded by *Hsa‐CBX3*) in skin fibroblast cell lines from Hutchinson‐Gilford progeria syndrome	[289]
	Mesenchymal stem cells (MSCs) from older individuals have reduced H3K9me3, HP1α (encoded by *Hsa‐CBX5*) and WRN RecQ‐like helicase (mutation of the *Hsa‐WRN* gene is causal of the Werner premature aging and DNA damage syndrome OMIM 277700) MSCs differentiated from embryonic stem cells (ESCs) with targeted knockout of the *WRN* gene's enzymatic domain had reduced global H3K9me3 WRN protein associated with HP1α and nuclear lamina‐heterochromatin anchoring protein LAP2β (produced by alternative splicing of *Hsa‐TMPO*), and with the H3K9 methyltransferase, SUV39H1 encoded by *Hsa‐SUV39H1*) Targeted knock‐in of catalytically inactive *SUV39H1* in wild‐type MSCs accelerated cellular senescence, similarly to *WRN*‐deficient MSCs	[290]
	Senescent—but not quiescent‐ human fibroblasts form heterochromatic foci, with enrichment of H3K9me3 and recruitment of HP1 proteins Foci formation depended on the transcriptional repression of E2F target genes by Rb (encoded by *Hsa‐RB1*) binding	[114]
	During replicative aging (late cell passage) of lung fibroblast cell line, telomeric destabilization and associated DNA damage response was accompanied by reduced H3K9me2 and H3K9me3, with concomitant H3K9me1 increase at telomeric chromatin	[291]
	Senescence associated heterochromatin formation (SAHF) involves the recruitment of H3K9me2/3 and HP1α, as well as the enrichment of histone variant macroH2A and the DNA damage marker, phosphorylated H2AX (γH2AX)	[114, 115, 116, 117, 118]
	H3K27me3	
*Drosophila*	H3K27me3 increased and broadened regions (AU: what regions? ED) with age, especially in the head Reduction of H3K27me3 by the deletion of PRC2 genes correlates with up‐regulation of glycolytic genes and increased lifespan The gene‐edited deletion of specific PRC2 components encoded by *Dme‐esc*, *Dme‐E(z)*, *Dme‐Pcl*, *Dme‐Su(z)12*, and PCR1 component *Dme‐Su(z)2* increased lifespan	[149]
	Increased H3K27me3 with age in the head, which was alleviated in *Dme*‐*mir‐34* mutants *miR‐34* targets PRC2 components.	[228]
	Increased H3K27me3 with age. Heterozygous mutations in the core subunits of PRC2, *Dme‐E(z)*, and *Dme‐esc*, increased longevity Mutations in the PR‐silencing antagonist, *trithorax Dme‐trx* suppressed the H3K27me3 elevation/longevity effect of *Dme‐E(z)* mutation *Dme‐E(z)* mutants showed stress resistance phenotypes and de‐repression of well‐characterized PC‐target gene *Dme‐Abd‐B*, and metabolic stress resistance gene, *Dme‐Odc1*	[150]
	*Dme‐E(z)* heterozygous mutants have sex‐specific extension of lifespan and healthspan (resistance to hyperthermia, oxidative stress, and endoplasmic reticulum stress; enhanced fecundity) Transcriptome profiling of *Dme‐E(z)* heterozygous mutants found altered expression of 239 genes involved with metabolism, immune response, cell cycle, and ribosome biogenesis	[151]
Human	Increased bulk H3K27me3 in HSCs and progenitor cells with age	[152]
	Reduced H3K27me3 and PRC2 enrichment at the *CDKN2A* (*INK4/ARF*) locus results in transcriptional activation, followed by events leading to senescence and SAHF formation	[159]
	*Histone methylation—transcriptional activation marks*	
	H3K4me3	
*Drosophila*	Genome‐wide overall increase in H3K4me3 peaks in aged flies	[91]
	H3K4me3 demethylase *Dme*‐*lid* mutants had male‐specific reduced‐lifespan	[168]
Human	Genome‐wide H3K4me3 assessment found that neurons isolated from prefrontal cortex had ~600 loci with H3K4me3 peaks in samples from infants compared to ~100 loci in old adults (>60 years) The H3K4me3 peaks that were specific to infant samples consisted mainly of genes involved with neurogenesis, neuronal growth, and differentiation genes, that suggest cellular plasticity	[292]
	H3K36me3	
*Drosophila*	Genome‐wide overall increase in H3K36me3 peaks in aged flies	[91]
Human	Blood DNA from individuals with Sotos syndrome (OMIM 117550), harboring *Hsa‐NSD1* H3K36 methyltransferase loss‐of‐function mutations, showed accelerated DNA cytosine methylation aging “clock” signature	[169]
	Histone acetylation	
*Drosophila*	Midlife (premortality plateau) phenotypes—increased oxygen consumption, reduced histone deacetylase inhibitor sensitivity, increased ATP citrate lyase (encoded by *Dme‐ATPCL*) activity, leading to elevated acetyl‐CoA associated with increased histone acetylation, and transcriptional alterations Decreasing *Dme‐ATPCL* activity, or decreasing H4K12‐specific acetyltransferase: *Dme‐chm*, alleviated aging‐associated changes and increased longevity	[293]
	Dietary restriction conditions known to extend lifespan via *Dme‐Sirt2* histone deacetylase‐mediated effects also delayed the age‐associated increase in TE transcription in the head and fatbody *Dme‐Sirt2* overexpression repressed TE elevation in the head and fatbody seen during aging	[93]
	Sirtuin‐activating compounds, resveratrol, and fisetin increased lifespan and prolonged fecundity. The longevity effect depended on functional *Dme‐Sirt2*	[73]
	*Dme‐Sirt2* overexpression increased lifespan *Dme‐Sirt2* downregulation blocked the lifespan‐extending effect of caloric restriction and *Rpd3* mutation	[71]
	Sirtuin‐activating compounds, alkylresorcinols, extended lifespan The lifespan extension depended on functional *Dme‐Sirt2*	[72]
	Strong dose‐dependent effect of *Dme‐Sirt2* overexpression (when two to fivefold overexpressed) on increasing lifespan *Dme‐Sirt2* overexpression increased the expression of the Puc protein phosphatase (encoded by *Dme*‐*puc*) and the heatshock response gene, Dme‐*DnaJ‐H* Puc protein phosphatase operates in a JNK signaling pathway	[174]
	Caloric restriction and *Dme‐HDAC1* (*Rpd3*) heterozygous loss‐of‐function mutation increased lifespan	[176]
	Ubiquitous *Dme‐Mi‐2* RNAi knockdown increased lifespan Mi2 is a component of the NuRD chromatin remodeling complex that includes HDAC1 and HDAC2 and thus the observed effects may be related to histone acetylation	[179]
Human	*Dme‐SIRT3* intronic enhancer polymorphism was found to be associated with old age, in which the allele that results in no enhancer activity was absent in >90 year old males A later meta‐analysis found inconclusive results	[294]
	*Dme‐SIRT3* polymorphism was associated with elderly survivorship	[295]
	Chromatin remodeling	
	*NuRD*	
*Drosophila*	Ubiquitous *Dme‐Mi‐2* RNAi knockdown increased lifespan	[179]
Human	Reduced expression of NuRD components in Hutchinson‐Gilford progeria syndrome (HGPS, OMIM 176670), HGPS‐derived cells and those from older donors Reduced HDAC1 activity (encoded by *Hsa‐HDAC1*) was also found (the NuRD chromatin remodeling complex that includes HDAC1 and HDAC2), suggesting that the mechanism could also be related to histone acetylation	[296]

**TABLE 3:** Conserved mechanisms in noncoding RNAs, RNA modification, and RNA editing implicated in aging in *Drosophila* and human

Species	Molecular mechanism and aging phenotypes	Species‐Gene	Concepts	References
	*RNAi pathway*			
*Drosophila*	Loss‐of‐function mutations *Argonaute 2* (*AGO2*) decreased lifespan Also increased TE expression in the brain, and memory impairment	*Dme‐AGO2*	Determination of adult lifespan; transposable element; gene expression; learning; or memory	[140]
	*Dcr‐2 (Dicer‐2*) overexpression increased lifespan and repressed TE elevation in the head and fatbody seen during aging *Dicer‐2* loss‐of‐function mutation increased DNA double‐strand breaks Reverse transcriptase inhibitor 3TC treatment attenuated TE activation and increased life span in *Dicer‐2* mutants	*Dme‐Dcr‐2*	Determination of adult lifespan; DNA double strand breaks; transposable element retrotransposon; gene expression	[93]
	*Mt2* (*Dnmt2* ortholog) overexpression increased lifespan	*Dme‐Mt2*	Determination of adult lifespan; gene expression	[141]
	tRNA methylation by *Dnmt2* and fragmentation during heatshock was shown to be required for efficient *Dcr‐2* function in *Drosophila* and thus the lifespan effect may be relevant to RNAi pathway	*Dme‐Mt2* *Dme‐Dcr‐2*	Determination of adult lifespan; gene expression; RNAi; heat shock	[297]
	*PIWI pathway*			
*Drosophila*	*piwi* attenuated intestinal stem cell activity reduction during aging Loss‐of‐function *piwi* in intestinal stem cells induced *Gypsy* retrotransposon transcriptional activation and insertion, DNA damage and apoptosis	*Dme‐piwi*	Intestinal stem cells; DNA damage response; apoptotic process	[146]
Human	Multiple studies have found many different PIWI‐interacting RNAs (piRNAs) to be relevant to human cancers	Many different piRNAs	Gene expression; cancer	Reviewed in [147]
	*Micro‐RNAs (miRNAs)*			
	*mir‐34*			
*Drosophila*	Loss of *mir‐34* resulted in gene profile alterations associated with accelerated brain aging and reduced lifespan Upregulation of *mir‐34* increases lifespan and alleviates neurodegeneration induced by human pathogenic polyglutamine disease protein	*Dme‐mir‐34*	Determination of adult lifespan; polyQ models neurodegenerative disease	[41]
	Age‐associated *mir‐34* reduction and its effect on brain aging was mediated by translational repression of *Eip74EF* and putatively via developmental gene silencing	*Dme‐mir‐34* *Dme‐Eip74EF*	miRNA mediated inhibition of translation	[227]
	miR‐34 RNA inhibits polycomb repressive complex 2 to modulate chaperone expression and promote healthy brain aging H3K27me3 levels increase with age and alleviated in *mir‐34* mutants	*Dme‐mir‐34*	ESC‐E(Z) complex H3K27me3	[228]
Human	*MIR34A* overexpressed with aging *SIRT1* gene is downregulated in PBMCs	*Hsa‐MIR34A* *Hsa‐SIRT1*	Gene expression PBMC	[229]
	GWAS association	*Hsa‐SIRT1* rs7096385‐**T**	GWAS on atrial fibrillation	[298]
	Type 2 diabetes is associated with increased expression of *MIR34A* in the heart	Hsa‐*MIR34A*	Type 2 diabetes mellitus gene expression Cardiac muscle tissue	[230]
	Overexpressed *MIR34A* in the lungs of patients with idiopathic pulmonary fibrosis, and functional studies found it promotes senescence and reduces cell proliferation	*Hsa*‐*MIR34A*	Gene expression Idiopathic pulmonary fibrosis Lung Cell senescence Cell proliferation	[231]
	Increased *MIR34A* expression was associated with advanced hepatocellular carcinoma	*Hsa*‐*MIR34A*	Gene expression Hepatocellular carcinoma	[232]
	*miR‐125*			
*Drosophila*	Loss of *miR‐125* results in reduced lifespan, climbing activity, and increased vacuoles in the brain	*Dme‐miR‐125*	Determination of adult lifespan	[31]
Human	Several studies have shown dysregulation of *MIR125b* (mature sequence of the *MIR125B1* and *MIR125B2* stem loop transcripts) in various cancer, and it appears to have tumor suppressive and oncogenic activity depending on tumor type	*Hsa‐MIR125B1* *Hsa‐MIR125B2*	Tumor suppressor gene oncogene	Reviewed in [234]
	*miR‐9*			
*Drosophila*	The level of *Dme‐miR‐9a* expression is elevated in testes germline stem cells *miR‐9a* targets transcripts encoding N‐cadherin, adhesion molecule *miR‐9a*‐null mutants can maintain larger number of stem cells despite aging, however, have decreased tissue regeneration and reduced fertility	*Dme‐miR‐9a* *Dme‐CadN* *Dme‐CadN2*	Gene expression male germ‐line stem cell population maintenance tissue regeneration fertility	[233]
Human	*MIR9* (mature sequence of the *MIR9‐1*, *MIR9‐2*, and *MIR9‐3* stem loop transcripts) expression increases with aging and *SIRT1* gene is downregulated in PBMCs Functional assay found that *miR‐9* interacts with the 3′UTR of SIRT1 mRNA	*Hsa‐MIR9‐1* *Hsa‐MIR9‐2* *Hsa‐MIR9‐3* *Hsa‐SIRT1*	Gene expression PBMC miRNA mediated inhibition of translation	[229]
	*MIR9* expression increases and IGF‐1R and FOXO1 are downregulated, in PBMCs	*Hsa‐MIR9‐1* *Hsa‐MIR9‐2* *Hsa‐MIR9‐3* *Hsa‐IGF1R* *Hsa‐FOXO1*	Gene expression PBMC	[235]
	*Long non‐coding RNAs (lncRNAs)*			
*Drosophila*	Relevance of lncRNA in aging is suggested by a study that profiled lncRNAs in the head, gut and fat body of flies reared in dietary restrictive condition that was found to increase lifespan	102 differentially expressed lncRNAs	Gene expression Response to caloric restriction Determination of adult lifespan	[224]
Human	lncRNA involved in aging lncRNA involved in aging‐related diseases	15 lncRNAs, mainly associated with senescence and cell cycle regulation		Reviewed in [213, 217]
	*Circular RNAs (circRNAs)*			
*Drosophila*	Accumulation of circRNAs in neural tissues to occur with aging		circRNA Drosophila neuron	[240]
	Strong increase of circRNAs during aging in photoreceptors		circRNA Drosophila photoreceptor cell	[241]
Human	Several lines of evidence collectively suggest a key role for circRNAs in aging		circRNA	Reviewed in [242]
	*RNA methylation—tRNA m* ^*5*^ *C*			
*Drosophila*	*Mt2* (*Dnmt2*) overexpression increased lifespan	*Dme‐Mt2*	Gene expression Determination of adult lifespan	[141]
	*Mt2* (*Dnmt2*) was found to be an tRNA m^5^C methyltransferase and thus the lifespan effect may be relevant to tRNA methylation	*Dme‐Mt2*	tRNA methyltransferase	[204, 205, 206, 207]
Human	Knocking down *TRDMT1* (*Dnmt2)* in human fibroblasts result in increased oxidative stress, DNA damage, upregulation of miRNAs related to proliferation, and results in senescence	*Hsa‐TRDMT1*	tRNA methyltransferase Fibroblast Oxidative stress‐induced premature senescence	[258]
	*RNA editing—Adenosine‐to‐inosine editing*			
*Drosophila*	Adenosine deaminase acting on RNA (*ADAR*) extended lifespan, with increased levels of histone modifications associated with heterochromatic silencing in brain neuronal cells	*Dme‐ADAR*	Determination of adult lifespan	[259]
Human	18 SNPs in *ADARB1* (5 SNPs) and *ADARB2* (13 SNPs) are associated with extreme longevity in a cohort combining the New England Centenarian Study and Southern Italian Centenarian Study. They were replicated in the Ashkenazi Jewish Centenarian Study and Japanese Centenarian Study	*Hsa‐ADARB1* *Hsa‐ADARB2*	Bayesian approach GWAS Determination of adult lifespan	[260]
	GWAS SNP interaction association to age related hearing impairment	*Hsa‐ADARB2* rs787274‐**?** x rs11250795‐**?**	Age‐related hearing impairment	[299]
	GWAS association to Alzheimer diagnosis accelerated after diagnosis of cognitive impairment	*Hsa‐ADARB2* rs10903488‐**?**	GWAS on Alzheimer's disease, cognitive decline measurement, and cognitive impairment	[300]
	GWAS association to age at menarche	*Hsa‐ADARB2* rs1874984‐**C**	GWAS on age at menarche	[301]
	GWAS association to age at menarche	*Hsa‐ADARB2* rs1874984‐**?**	GWAS on age at menarche	[302]

**TABLE 4:** Conserved mechanisms involving nuclear and chromosomal architecture implicated in aging in *Drosophila*, mouse, and human

Species	Nuclear and chromosomal architecture	References
	Maintenance of meiotic sister chromatid cohesion	
*Drosophila*	Increased oocyte meiotic segregation errors and nondisjunction with heterozygous mutation in cohesin subunit *Dme‐SMC1* with aging.	[303]
	Increased susceptibility to aging‐related meiotic segregation errors and nondisjunction with heterozygous mutation in *Dme*‐ord with reduction of centromere‐proximal heterochromatin.	[304]
Mouse	Female mice deficient in the meiosis‐specific cohesin SMC1β encoded by *Mmu‐Smc1b* show nondisjunction and age‐dependent loss of meiotic sister chromatid cohesion	[305]
Human	Downregulation of the meiosis‐specific cohesin subunits encoded by *Hsa‐REC8* and *Hsa‐SMC1B*, in women aged 40 and over compared with 20 years.	[306]
	Multiple lines of evidence suggest decreased meiotic sister chromatid cohesion with age and support that it plays key roles in the maternal age effect on meiotic segregation.	Reviewed in [307, 308]
	Integrity of the nuclear lamina	
*Drosophila*	Aging‐related progressive reduction in lamin‐B (encoded by *Dme‐Lam*) in the fat body was found to contribute to chronic inflammation and gut hyperplasia. Depletion of lamin‐B in the young/larval fat body via RNAi *Dme‐Lam* results in reduced amount of heterochromatin, and increase in retrotransposon expression and DNA damage	[132, 133, 134]
Human	Hutchinson‐Gilford progeria syndrome (HGPS, OMIM 176670), characterized by premature aging phenotype, is caused by various germline mutations in *Hsa‐LMNA* encoding lamin A/C.	[129, 130]
	In senescent dermal fibroblasts and keratinocytes, *Hsa‐LMNB1* encoded lamin‐B1 protein expression is reduced. *Hsa‐LMNB1* overexpression induces senescence.	[131]
	Lamina‐associated domains (LADs) are genomic regions that form molecular contacts with nuclear lamins. LADS have many of the properties of heterochromatin at the periphery of the cell nucleus.	[309]

### 3.1 Heterochromatin redistribution and epigenetic changes during aging

#### 3.1.1 Overview

The eukaryotic genome is highly organized with nucleosomes (DNA with histone proteins, H2A, H2B, H3, and H4) as a fundamental unit of chromatin. The histone proteins with various types of chemical modifications are highly conserved across species.^86^ Chromatin consists of heterochromatin and euchromatin regions.^86^, ^87^, ^88^ Heterochromatin are gene‐poor, transcriptionally silent, and highly condensed regions, which are generally characterized by histone hypoacetylation and the enrichment of repressive histone marks, H3K9me2 and H3K9me3, and HP1α proteins.^86^, ^88^ In contrast, euchromatin regions are relatively gene‐rich, transcriptionally active, less condensed, and enriched with histone hyperacetylation and active histone marks such as H3K4me2 and H3K4me3.^86^, ^88^

Heterochromatin redistribution in the aging process has been widely reported. While an aberrant gain of heterochromatin at specific gene promoters induces transcriptional repression, in other promoters, the loss of heterochromatin results in abnormal transcriptional activity. It was speculated that the loss of protective heterochromatin at constitutive heterochromatin loci during the aging process might increase the rate of chromosomal aberrations and the susceptibility to double‐stranded DNA breaks in the genome.^89^, ^90^ Another notable consequence is the activation of transposable elements (TEs) that can cause mutagenesis events such as insertion and deletion.^89^, ^90^ Identifying the role of heterochromatin in specific regions has therefore become one of the major goals in the aging field.

#### 3.1.2 Repressive hallmark of heterochromatin—H3K9 methylation and HP1a

Generally, aging‐related disruptions in the repressive marks, H3K9me3 and HP1a, especially in constitutive heterochromatin, are correlated with reduced lifespan and compromised healthspan measures in *Drosophila*.^32^, ^51^, ^91^, ^92^, ^93^, ^94^, ^95^ Specifically, a study^91^ found that these marks were most dramatically reduced at pericentric heterochromatin and the fourth chromosome in aged flies. Another study in *Drosophila* demonstrated that the loss‐of‐function *HP1a* heterozygous mutants with loss of heterochromatin resulted in the disruption of ribosomal RNA (rRNA) gene loci and an increase in ribosomal RNA (rRNA) transcription, and these flies exhibited significantly shortened lifespan and the loss of muscle integrity, thus demonstrating a link between the loss of heterochromatin and metabolism.^32^ It was also shown in this study that overexpressing *HP1a* had the opposite effects, resulting in increased lifespan and the maintenance of muscle integrity.^32^ Additionally, knocking down *HP1a* or *Su(var)3‐9* (H3K9 methyltransferase) in the male germ cells caused an early reduction of stem cells^51^; stem cell exausting is widely regarded as one of aging phenotypes. Together, these studies strongly support that H3K9 methylation and HP1a play an important role in maintaining the heterochromatin, and the disruption of heterochromatin can negatively impact both lifespan and healthspan.

Other pathways were found to regulate heterochromatin through their interaction with HP1 and Su(var)3‐9. A genetic screen in *Drosophila* that intended to identify novel components in the Janus kinase (JAK) signaling pathway, found that *HP1* and *Su(var)3‐9* mutations enhanced a hyperactive *JAK* allele, which globally disrupts heterochromatic gene silencing.^92^ It was demonstrated that a JAK downstream transcription factor, the signal‐transducer and activator of transcription protein at 92E (Stat92E), in its unphosphorylated form, interacts with HP1a, promoted heterochromatin formation,^92^, ^96^, ^97^ and enhanced genome stability^98^; moreover, this interaction was also found to protect against leukemia‐like cancer formation.^92^ A similar mechanism was also demonstrated in mammals to suppress cancer development,^99^, ^100^ highlighting the advantage of using *Drosophila* for discovering conserved mechanisms.

These findings in *Drosophila* is consistent with an aging model in mammals,^101^, ^102^, ^103^ in which constitutive heterochromatin regions including TEs and rDNA also lose the major heterochromatin markers, HP1a and H3K9me3, while aberrant heterochromatin forms in other regions. The loss of heterochromatin during aging is conserved in budding yeast, mice, and human cells, underscoring the importance of this mechanism.^104^ Mobilization of TEs during the aging process has become increasingly appreciated.^105^ For example, TE activation and genomic instability were found in the neurons of a *Drosophila* model of AD as well as the postmortem brain tissue from AD patients.^106^ Recently, an in vivo drug screen in *Drosophila* identified methotrexate, a compound for treating arthritis, to augment H3K9me3 and HP1a foci, thus promoting heterochromatin formation and genome stability.^107^ This may lead to new interventions targeting aging. These findings highlight the relevance of the *Drosophila* as a model for understanding the aging process in humans.

During mammalian aging, prominent genomic regions acquire the aberrant heterochromatin including the Senescence‐associated heterochromatin foci (SAFH). This can be triggered by the upregulation of the p16(INK4a) tumor suppressor and the consequent repression of E2F‐target genes mediated by the Retinoblastoma (Rb) tumor suppressor.^108^, ^109^, ^110^, ^111^, ^112^, ^113^ The SAFH formation involves the recruitment of H3K9me2/3 and HP1α, the enrichment of a histone variant, macroH2A and a DNA damage marker, phosphorylated H2AX (γH2AX).^114^, ^115^, ^116^, ^117^, ^118^ Other pathways are also involved. It was speculated that senescent cells drive aging phenotypes by secreting signaling molecules, including those that promote chronic inflammation^119^; inflammation is widely accepted as an aging hallmark Replicative senescence, an irreversible arrest of cell proliferation perhaps due to DNA repairing, can also be triggered by p53^115^; DNA damages generally accumulate with aging. Studies of cellular senescence in the mammalian system are complicated because senescence occurs in progressive steps,^120^ and different types of senescent states appear to exist, which are caused by different triggers^121^ and involve different molecular pathways.^115^, ^122^

However, the *Drosophila* genome lacks the conservation of p16, which is one of the well‐established triggers of senescence and plays an important role in regulating aging in mammalian cells. Instead, overexpression of a hyperactive allele of the *Ras* oncogene, along with mitochondrial dysfunction, could induce senescence.^123^ The downstream events are more conserved, including the activation of p53 and JNK pathways and the inhibition of the Hippo pathway; and in turn, these events lead to the upregulation of the Unpaired (Upd) inflammatory cytokine, an aging hallmark Clonal analyses also found an increase in H3K9me3 in the cells in which senescence was induced. Therefore, the *Drosophila* model appears to have a mammalian‐like mechanism of cellular senescence. Future studies that address the regulation of specific loci subject to heterochromatin redistribution are important in both *Drosophila* and humans.

#### 3.1.3 Nuclear lamina and heterochromatin

Nuclear lamina (NL), a lining of the inner nuclear envelope, consists of filaments, lamins, and lamin‐associated proteins, and is directly tethered with heterochromatin.^124^ Heterochromatin domains of the genome are anchored to the NL, allowing them to reside in the nuclear periphery. It has been suggested that the spatial positioning of the heterochromatin is mechanistically important for their repressiveness of transcriptional activity.^125^ The lamin‐B receptor (LBR) forms a complex with HP1α, demonstrating a direct link.^126^ Moreover, Proline Rich 14 (PRR14), a protein that binds to lamin‐A (LMNA), can also interact with HP1α. In human cells, the PRR14 depletion results in the redistribution of H3K9me2 and H3K9me3, releasing chromatin from nuclear periphery to the interior area.^126^ The recent development of chromosome conformation‐capture technologies allows the assessment of 3D chromatin structure. Using this technique, it was found that aging‐associated heterochromatin redistribution and chromatin alterations also occurred at higher orders of the structure, suggesting a complex role played by chromosomal organization in senescence and aging.^127^, ^128^ Further understanding of chromatin organization and sub‐nuclear localization is expected to lead to important findings in the aging mechanism and aging‐related diseases.

The Hutchinson‐Gilford progeria syndrome (HGPS, https://www.omim.org/entry/176670), which is characterized by an extreme premature aging phenotype, is caused by various germline mutations in *LMNA*, suggesting a mechanistic link between nuclear architecture, heterochromatin, and aging.^129^, ^130^ In senescent human dermal fibroblasts and keratinocytes, lamin‐B1 (LMNB1) and lamina‐associated polypeptide 2 (LAP2) protein levels are reduced, and this reduction was also recapitulated in skin harvested from young vs old individuals.^131^

In *Drosophila*, studies have also provided evidences for the relevance of the NL in natural aging.^132^, ^133^, ^134^ A progressive reduction in LMNB expression in the fat body was observed with aging, and moreover, found to contribute to chronic inflammation and gut hyperplasia.^134^ Intriguingly, knocking down LMNB in young adult or larval fat body resulted in reduced heterochromatin, and an increase in retrotransposon expression and DNA damage.^132^, ^133^ Collectively, these studies provide evidence of the importance of NL in controlling nuclear architecture, heterochromatin, TE activation, genome instability and chronic inflammation in aging. This again suggests the conservation between *Drosophila* and human aging.

#### 3.1.4 RNAi pathway related to heterochromatin and TE silencing

One of the major roles of the RNAi pathway is in regulating heterochromatin‐induced silencing of repetitive DNA loci, such as TEs, pericentric satellite repeats, and telomeric repeats.^135^, ^136^, ^137^ (Its other major role in post‐transcriptional gene silencing is discussed in Section 3.3). The RNAi pathway's regulation of heterochromatin formation involves the interaction between RNA and H3K9me3 methyltransferases and promoting HP1α recruitment.^138^, ^139^ In *Drosophila*, RNAi pathway components, Dicer‐2 (Dcr‐2) and Agonaute (Ago2), have been shown to regulate lifespan and other aging‐related phenotypes by suppressing TEs.^93^, ^140^, ^141^ Overexpressing *Dcr‐2* in *Drosophila* led to increased lifespan, with concomitant repression of TEs, while the loss‐of‐function of *Dcr‐2* resulted in an increase in DNA double‐strand breaks.^93^ Lamivudine (3TC), a rreverse transcriptase inhibitor, prolonged lifespan and attenuated TE mobilization caused by *Dcr‐2* loss‐of‐function mutants.^93^ An *Ago2* loss‐of‐function mutation reduced lifespan and caused neuronal phenotypes such as memory impairment and TE activation in the brain,^140^, ^142^ and neurodegeneration and increased brain vacuoles (^142^; described in detail in Section 3.4). In humans, knocking down *DICER 1* in HEK293T and HepG2 cells increased double‐stranded DNA damage,^143^ however, the mechanistic studies in this area have not been conducted in *Drosophila*.

#### 3.1.5 PIWI/piRNA related to heterochromatin and TE silencing

It is well‐established that the PIWI/piRNA pathway plays a crucial role in silencing TEs in the germline of *Drosophila* ^144^ by regulating heterochromatin formation.^145^ More recently, a *Drosophila* study has found that Piwi is crucial for suppressing age‐related TE expression in intestinal stem cells and the maintenance of epithelial homeostasis, thus demonstrating its new role in somatic stem cell maintenance.^146^ The PIWI pathway has recently been appreciated as a key player in cancer as well.^147^

#### 3.1.6 Silencing histone methylation mark, H3K27me3

It is well‐established that H3K27me3 promotes gene silencing and is associated with polycomb repressive complex (PRC) 1 and 2.^148^ These complexes and sub‐complexes share some components and have distinct and overlapping target loci. Evidence in *Drosophila* supports the presence of specific genomic areas with aging‐associated augmentation of H3K27me3,^149^, ^150^, ^151^ and this increase is particularly high in the head.^149^ Loss‐of‐function mutations in the genes encoding specific PRC1 and PRC2 components suppressed the elevation of H3K27me3 during aging and improved lifespan and healthspan. Among PRC components, *Enhancer‐of‐zeste* (*E[z]*) mutants have been particularly well‐characterized.^150^, ^151^ Observed healthspan effects include improved stress resistance to hyperthermia, oxidative stress and endoplasmic reticulum stress, as well as enhanced fecundity.^150^, ^151^ The stress resistance was accompanied by the upregulation of many target genes including the stress resistance gene, *Ornithine Decarboxylase 1* (*Odc1*).^150^ Further genome‐wide transcriptome profiling of these animals found altered expressions of 239 genes that were mainly involved in metabolism, immune response, cell cycle, and ribosome biogenesis.^150^ Further characterization of the H3K27me3 regulation during aging may find potential targets for slowing down the aging in the epigenome.

In human studies, a bulk increase in H3K27me3 was also detected with aging, when assessing hematopoietic stem cells (HSCs) and progenitor cells.^152^ Moreover, DNA methylations at the CpG sites of the epigenetic clock model of human aging (described in detail in Section 3.2) are significantly correlated with Polycomb‐group (PcG) target loci.^153^ These observations are in line with the findings in *Drosophila* where H3K27me3 increases with natural aging. Cross‐talks between DNA methylation and H3K27me3 also occur in other contexts.^154^, ^155^, ^156^ As opposed to the *Drosophila* genome where PRCs are mainly recruited at Polycomb repressive elements (PREs), far fewer PREs have been described in the mammalian genome.^157^ One possibility could be that due to the lack of DNA methylation, PREs are increasingly important to guide PRCs in the *Drosophila* genome.

There are other loci that can be affected by augmented levels of H3K27me3.^158^ For example, a study in cell culture demonstrated that replicative senescence and SAFH formation resulted from a reduced level of H3K27me3 at the *CDKN2A* (*INK4/*ARF) locus.^159^ Metformin, an anti‐diabetic drug, was found to be a specific inhibitor of the H3K27me3 demethylase, Kdm6A,^158^ and the cells treated with Metformin had a global increase in H3K27me3. Therefore, metformin may be applicable as an anti‐aging intervention by inhibiting the H3K27me3 demethylation at the locus such as *CDKN2A*. However, it is critically important to note that inactivating this locus may drive oncogenesis, therefore, a balanced activity or expression is crucial.^160^, ^161^

#### 3.1.7 Gene activation marks, H3K4me3, and H3K36me3

In the study that found an overall decrease in the repressive H3K9me3 mark in aging *Drosophila*, the active marks, H3K4me3 and H3K36me3 showed an overall increase.^91^ H3K4me3 that located at the promoter regions are associated with transcriptionally active genes.^162^ H3K36me3 is enriched in the gene bodies, regulating transcriptional fidelity and alternative splicing events.^163^, ^164^ Studies demonstrated that both activating marks can antagonize H3K27me3,^165^, ^166^, ^167^ and the interplay between these histone marks might be important during aging. For instance, a mutation in the *trithorax* (*trx*) encoding a H3K4 methyltransferase was found to negate the lifespan increasing effect of *E(z)* mutation.^150^ Elucidating the loci related to these marks during aging, including the identification of the regulatory enzymes and other factors can help find the interventions to suppress aging‐related phenotypes. It is easy to conduct genetic studies in this area using the *Drosophila* model.

H3K4me3 and H3K36me3 appear to be also relevant to human aging brain. A genome‐wide H3K4me3 profiling found that in neurons isolated from infant prefrontal cortex, H3K4me3 peaks in several hundred loci, compared to approximately a hundred in samples from old people (>60 years).^168^ The H3K4me3 peak sites in infants consisted mainly of the genes involved in neurogenesis, neuronal growth, and differentiation. The link between H3K36 methylation and human aging was also observed in Sotos syndrome patients who had a mutation in a gene encoding H3K36 methyltransferase.^169^ Future studies of H3K4me3 in other tissues and H3K36me3 in natural aging are expected to yield informative results. Concomitantly, assessing transcript levels and alternative splicing isoforms would be useful.

#### 3.1.8 Sirtuin as an anti‐aging regulator

The *Sirtuin* family of NAD‐dependent histone deacetylases (HDACs) has been in the limelight in aging studies because its stimulation or overexpression has been shown as a promising avenue for anti‐aging.^170^, ^171^ Although it is well‐established that *Sirtuins* mediate the longevity effect of caloric restriction (diet condition studies are described in detail in Section 2.3), its role played in *Drosophila* lifespan has been controversial. Whereas one study reported no effect,^172^ some others observed lifespan extension, but only when the expression level of *Sirtuin 2* (*Sirt2*) was two to fivefold upregulated.^71^, ^173^, ^174^ Above this range, *Sirt2* was found to be detrimental, as evidenced by the triggering of the JNK negative feedback loop kinase, *puckered* (*puc*) and the heat‐shock stress response gene, *DnaJ homolog* (*dnaJ‐H*), as well as caspase‐mediated apoptosis.^174^, ^175^

Consistent with the idea that *Sirt2* overexpression can increase lifespan, Sirtuin‐activating compounds, resveratrol and fisetin were found to improve longevity and prolong fecundity when functional *Sirt2* was present.^73^ A later *Drosophila* study confirmed the effect of resveratrol, and further demonstrated that a mixture of Sirtuin‐activating compounds, alkylresorcinols, similarly extended lifespan in a *Sirt2*‐dependent manner.^72^ Taken together, these studies support that the lifespan enhancement effect of *Sirt2* is conserved across species including *Drosophila*, and that the level of its expression is functionally important. In *Drosophila*, *Sirt2* overexpression can repress the age‐related TE activation in the head and fat body,^93^ suggesting its potential link to H3K9me3‐HP1a and RNAi, suggesting its relevance to the lifespan extension involving Sirtuins in humans.

Different classes of histone deacetylases have been shown to work together while retaining distinct function. *Drosophila* mutants of the histone deacetylase, *HDAC1* are long‐lived.^176^ *HDAC1* is highly conserved across species and belongs to Class I—different from sirtuins that belong to Class III HDACs.^177^ *Sirt2* upregulation was found in *HDAC1* mutants,^176^ and the lifespan effect was similar to caloric restriction or other interventions that enhanced the level of *Sirt2*. Interestingly, caloric restriction did not extend the lifespan of the *HDAC1* mutants.^176^ How these two histone deacetylases interact requires further investigation.

Sirturins also regulate nonhistone targets. In both *Drosophila* and mammalian studies, p53 protein has been identified to be a major target of deacetylation by SIRT2.^178^

#### 3.1.9 Nucleosome remodeling regulators in aging

The interaction between the NuRD—HDAC complex and PRC might be important in regulation of nucleosome remodeling during aging across species. Increased lifespan was found in *Drosophila* with *Mi2* knockdown.^179^ Mi2 protein is a component of the nucleosome remodeling and deacetylase (NuRD) complex that also includes HDACs and conserved across species.^180^ This protein was initially found to participate in the PcG‐mediated repression at *Hox* clusters.^181^ The deacetylation of H3K27ac facilitates the methylation at the H3K27 residue, and this antagonistic mechanism was previously observed in both mammalian cells^182^ and in *Drosophila*.^183^ Ascertaining the age‐dependent effects of nucleosome remodeling vs HDAC‐dependent gene expression at NuRD target sites can help elucidate the interactions between these mechanisms. It can also aid in understanding their relevance to the heterochromatin marks such as H3K27me3.

### 3.2 . DNA methylation alteration during aging

#### 3.2.1 5‐methylcytosine

A recent advance in aging research, which has received extensive attention, is the development of the “epigenetic clock” for measuring the biological age of tissues or cells.^153^, ^184^, ^185^, ^186^, ^187^, ^188^, ^189^ The clock is based on a linear regression model that correlates the changes of 5‐methylcytosine (5mC) marks at specific CpGs with the age. The age‐dependent alteration of DNA methylation in the clock appears to be conserved in mice, chimpanzees and other organisms. Using the clock, liver samples obtained from obesity patients were found to have accelerated epigenetic age.^190^ It has been informative to evaluate the effect of different lifestyle factors and other interventions on aging,^191^ as well as applying the model longitudinally.^192^, ^193^

In *Drosophila*, *C elegans*, and yeast species, the presence of 5mC in the genome has been long debated.^194^, ^195^, ^196^, ^197^ In *Drosophila*, 5mC was found relatively more abundantly in early embryos,^195^, ^198^ but with a low level in adult tissues.^195^, ^197^, ^198^, ^199^, ^200^, ^201^, ^202^, ^203^ In its genome, only one methyltransferase, *Methyltransferase 2* (*Mt2*, previously named as *DNA methyltransferase* [*Dnmt2*]) is present with a mammalian orthologue. It lacks the conservation with the human de novo DNA methyltransferases (DNMTs), *Dnmt3A/B*, and the maintenance DNMT, *Dnmt1*. *Mt2/Dnmt2* was initially named as a DNMT, but later found to act predominantly as a tRNA m^5^C methyltransferase,^204^, ^205^, ^206^, ^207^ although in the context of tRNA‐DNA hybrids, it can also act on DNA.^208^ In *Drosophila*, the overexpression of *Mt2/Dnmt2* increases lifespan, however the mechanism remains elusive.^141^ The minimal presence of 5mC in adult flies suggest that it likely does not mimic methylation changes in 5mC in human aging, thus not a suitable model for the aging clock.

#### 3.2.2 6‐methyldeoxyadenosine

It was found recently that *N^6^*‐6‐methyldeoxyadenosine (6mA) is another DNA methylation mark that is abundantly present in both the human^209^ and *Drosophila* genome.^210^ In *Drosophila*, 6mA was demonstrated to be regulated by DNA 6mA demethylase (DMAD), and its presence was correlated with TE expression in the ovary.^210^ A different study showed that 6mA transcriptionally regulated *zelda* via the recruitment of Jumu, a 6mA reader, to facilitate the maternal‐to‐zygotic transition.^211^ It was also demonstrated that 6mA might dynamically regulate the genes involved in neurodevelopment and neuronal functions and TE activity in the brain.^212^ However, the role of 6mA in aging is still lacking in any organism. Given the relevance of TE in aging,^105^ 6mA and its regulators warrant further investigation for its role as a functional substitute for 5mC and a potential aging clock in *Drosophila*. It is also of great interest to delineate the similarities and differences between the mechanistic roles of 6mA and 5mC.

### 3.3 Noncoding RNAs

The transcriptional dysregulation due to chromatin alterations has been shown to affect both protein‐coding gene transcripts and noncoding RNAs. Studies of different noncoding RNAs involved in *Drosophila* aging, and their relevance to human aging are summarized in Table 2. The RNAi and Piwi/piRNA pathways, relevant to small noncoding RNAs, has been discussed in relation to heterochromatin in Section 3.1.

#### 3.3.1 Long noncoding RNAs (lncRNAs)

LncRNAs can act as an enhancer to facilitate transcription, as well as a decoy to prevent or guide the recruitment of transcription factors and chromatin modifying factors, and the interactions between DNA methylation or histone modification mechanisms and lncRNAs are evident.^213^ For instance, *MALAT1* lncRNA, which was found to be upregulated in various cancer types in humans,^214^ can regulate the recruitment of histone‐lysine N‐methyltransferase enzyme, EZH2 to promote H3K27 methylation at specific genomic loci.^215^ LncRNAs can also act as a scaffold to promote the formation of protein complexes, which regulate various processes such as transcription, alternative splicing, translation, rRNA maturation, microRNAs binding to their target mRNAs, and signaling molecule phosphorylation.^213^ Moreover, they can serve as a precursor to small RNAs.^213^ A plethora of other studies have linked noncoding RNAs to human aging^216^, ^217^ and aging‐related diseases^213^ including cancer,^218^, ^219^, ^220^ cardiovascular,^221^ and neuronal aging^222^ and metabolism and cellular senescence.^223^

LncRNAs have not been investigated extensively in the context of aging in *Drosophila*. A study profiled lncRNAs in the head, gut and fat body of flies reared in the lifespan‐extension dietary restrictive condition and identified 102 differentially expressed lncRNAs.^224^ Bioinformatic analyses indicated that these lncRNAs mainly regulated the metabolism pathways such as mTOR, foxo, and Wnt signaling pathways, which have been implicated in aging across species including humans. Such profiling studies can be highly informative guiding the investigation on the targets of identified lncRNAs. Given that a high level of conservation of major lncRNAs have been noted,^225^, ^226^ studies in *Drosophila* are expected to lead to mechanistic insights.

#### 3.3.2 Small noncoding RNAs

In a *Drosophila* Hungtington's disease model that overexpressed the human pathogenic polyglutamine disease protein, a knockout in *miR‐34* resulted in reduced lifespan with accelerated brain aging, whereas its upregulation extended lifespan and alleviated neurodegeneration.^41^ The Ecdysone‐induced protein 74EF gene, *Eip74EF* was found to be a target of *miR‐34* and upregulated in the *miR‐34* deletion strain.^41^ Another study confirmed the disruption of the Ecdysone signaling in *miR‐34* knockouts and reported a defect in innate immune response.^227^ These observations suggest a new role of the Ecdysone pathway in aging. Another study showed that *miR‐34* targeted PRC2 components, *Polycomblike* (*Pcl*) and *Su(z)12*, and that in *miR*‐34 deletion animals, the H3K27me3 accumulated in the brain and the gene expression profile was associated with advanced aging.^228^ Also, in this study, *miR‐34* upregulation was shown to lead to the alleviation of neurodegeneration induced by pathogenic polyglutamine protein overexpression.^228^

The miR‐34 family is conserved across species including humans. The human *miR‐34a* (member of the miR‐34 family) was found to be upregulated with aging in peripheral blood mononuclear cells (PBMCs) and target *SIRT* genes.^229^ Given the importance of maintaining the level of Sirtuins (described in part 3b.iv. above), targeting *miR‐34a* may be a feasible anti‐aging intervention. Other studies have also shown the relevance of *miR‐34a* to cancer and diabetes.^230^, ^231^, ^232^ These observations underscore the importance of *miR‐34a* in aging across species.

Other miRNAs relevant to aging include *miR‐125* and *miR‐9a*. The loss of *miR‐125* in *Drosophila* was found to reduce lifespan, impact climbing activity, and increase neurodegeneration in the brain.^31^ *miR‐9a* was found to regulate the maintenance of male germline stem cell.^233^ The human homologs of these miRNAs have been implicated in aging and cancer in humans.^229^, ^234^, ^235^ Identifying other miRNAs involved in aging may yield new putative targets to counter aging phenotypes.

Age‐related chromatin alterations can affect the expression of various miRNAs, as shown by some examples above. MiRNAs have also been shown to also downregulate DNMTs to promote aging‐related cancer progression.^236^, ^237^, ^238^, ^239^

#### 3.3.3 Circular RNAs

Circular RNAs (circRNAs), a new species of small RNAs have emerged as being important in regulating aging In *Drosophila*, the age‐related accumulation of circRNAs was evident in the brain and photoreceptors.^240^, ^241^ Further mechanistic studies in *Drosophila* are expected to be informative, especially since circRNAS have been increasingly appreciated in human aging.^242^

### 3.4 Emerging roles of RNA methylation and RNA editing

RNA methylation and other types of chemical modifications such as hydroxylmethylation and pseudouridylation exist abundantly.^243^, ^244^ Although the first RNA methylation mark, *N* ^*6*^‐Methyladenosine (m^6^A) was found in 1970's, the regulators (writers and erasers) and the effectors (readers) have begun to be understood recently.^243^, ^245^ The abbreviation, m^6^A, is used for RNA modification, distinguishing from 6mA for DNA modification.^246^ RNA methylation (m^6^A and other types) has been shown to regulate the stability of various RNA species, mRNA alternative splicing, translation, nuclear‐cytoplasmic shuttling, and interactions between different RNA species. The writers, erasers, and readers of m^6^A have been shown to be conserved across species including *Drosophila* and humans.^247^

Although m^6^A has not been directly implicated in aging in *Drosophila*, phenotypes that are loosely relevant have been found. For example, the loss‐of‐function mutations in m^6^A regulators result in the aging‐like defects in neuronal activity and oogenesis.^248^, ^249^ The high expression of the m^6^A methyltransferase complex during early embryogenesis suggest that these aging phenotypes likely arise during early development rather thanthe aging process. Thus, evaluating m^6^A profile changes, or a temporal knockdown (presented in Section 2.1) of m^6^A regulators later in life may help discover the links between m^6^A and aging. In a human study profiling m^6^A‐containing transcripts in peripheral blood mononuclear cell (PBMCs), it was found that the overall enrichment level of m^6^A decreased in old people, compared to young people.^250^ Specifically, for *AGO2* mRNA, both its abundance and the m^6^A enrichment were reduced. m^6^A was also found to regulate other biological processes relevant to aging in humans, including self‐renewal in stem cells,^251^ the circadian rhythm,^252^ promoting cancer stem cells,^253^ and enhancing tumorigenicity.^254^

Other significant RNA modifications including 5‐methylcytosine (m^5^C), *N* ^*6*^,2′‐*O*‐dimethyladenosine (m^6^A_m_), 2′‐*O*‐methylation (2′Ome), and pseudouridylation might be also involved in aging.^255^, ^256^, ^257^ As mentioned above in Section 3.2, in *Drosophila*, the overexpression of *Mt2/Dnmt2*, which acts mainly as a tRNA m^5^C methyltransferase,^204^, ^205^, ^206^ increases lifespan.^141^ Conversely, knocking down *TRDMT1*/*Dnmt2* in human fibroblasts increased oxidative stress, DNA damage, miRNAs targeting transcripts related to proliferation, resulting in senescence.^258^ In *Drosophila*, 2′Ome becomes enriched in specific miRNAs with age.^142^ The study also reported that more miRNAs become associated with Ago2, but not with Ago1 during aging.^142^ Indeed, *Ago2* mutation led to decreased lifespan and neurodegeneration accompanied with increased brain vacuoles.^142^ Thus, further studies of RNA modification mechanisms and their relation to the Ago2 loading in the context of aging may be important.

In addition to RNA base modifications, RNA editing has been recently described to play a role in aging. An interplay between RNA m^6^A modification and RNA adenosine‐to‐inosine (A‐to‐I) base editing was found; the presence of the two alterations appeared to be negatively correlated.^243^ In *Drosophila*, a hypomorphism of the adenosine deaminase acting on RNA (*ADAR*) gene, which encodes an A‐to‐I editor, was found to cause the extended lifespan. This was accompanied in neurons with increased levels of histone modifications that are associated with heterochromatic silencing.^259^ Human cohort studies found single nucleotide polymorphisms (SNPs) in the orthologues, *ADARB1* and *ADARB2* to be associated with longevity,^260^ suggesting the clinical relevance of the findings in *Drosophila*. In addition, various recent studies have also linked the misregulation of RNA editing to the development of cancers and metabolic disorders.^261^

## 4 CONSIDERATIONS FOR FUTURE STUDIES OF AGING

### 4.1 Tissue specificity, single‐cell methods, ‐omics, and integrative approaches

Aging‐related epigenetic alterations and gene expression changes are distinctive in different tissues and cell types, demonstrated by epigenetic aging clocks.^153^, ^262^, ^263^ Additional studies to identify other biomarkers through transcriptomic, proteomic and metabolomic profiling with multi‐tissue approach may lead to the identification of molecular changes across different tissues and cells and further improve the epigenetic clocks. Recent development for single‐cell DNA methylation analysis^264^ is expected to yield deep insights in epigenetic drift mechanisms and human aging‐related heterogeneity with high resolution.^191^ The trending approach, not just in the aging field, but widely, involves emerging technologies that allow molecular signature assessment of single cells, in addition to bulk tissues. The obvious limitation for the human studies is that not all tissues and cells can be harvested for investigation.

Moreover, an integrative ‐omics obtaining information from a wide variety of types of molecular signatures (genomics, epigenomics, transcriptomics, proteomics, metabolomics, metagenomics, and other ‐omics) is an emerging approach that provides comprehensive and unbiased information. ^265^, ^266^, ^267^ It would invariably increase the accuracy of predicting the aging development and improve our understanding of the underlying molecular mechanisms. It is particularly powerful, if combined with rich genetics in *Drosophila*. It is clear from animal models that it is important to consider genetic backgrounds for aging research, which presents a challenge in human studies in which heterogeneity is a given.

Applying machine learning algorithms to select panels of predictors and construct regression models for estimating biological age, or for risk profiling of a specific aging‐related outcome, could potentially be conducted using samples from geriatric patients as well as model organisms including *Drosophila*. Moreover, such algorithms would provide additional clinical research tools for standardizing populations or measuring the success of therapeutic interventions. Another type of analyses is to identify differentially expressed biomarkers and monitoring them longitudinally. Such information could be used for elucidating molecular mechanisms underlying the aging process. Network analyses of the relevant factors and identifying central nodes would aid the identification of effective targets and development of pharmacological interventions, and this is particularly useful for *Drosophila* and other model organisms that have rich network information available. The contribution of the microbiome and microvirome to aging remains to be largely elusive and the efforts including metagenomics is highly in demand. Developing computational and visualization pipelines for integrative‐omics approaches are challenging because it requires the consideration of various molecular types with different scales and formats to high dimension and is an on‐going effort.^268^, ^269^, ^270^, ^271^, ^272^

### 4.2 Other uses of the *Drosophila* system in aging research

As indicated in Table 1, *Drosophila*, with the plethora of healthspan phenotypes that are relevant to human aging, has been shown to contribute significantly to the aging studies in vivo. It would also be advantageous for meticulous quantification of “proportional” vs “chronological” healthspan extension, which is not easy in human studies.

*Drosophila* have been used extensively as a model to elucidate fundamental mechanisms of all aspects of biology and there are still endless discoveries that could be made in this highly characterized genetic system. As we discussed, the limited presence of 5mC in the adult *Drosophila*, points to the skepticism over its being a major epigenetic mark for measuring aging and the importance of developing aging models based on other epigenetic marks. The abundance of 6mA in the *Drosophila* genome and the lack of studies on its possible role in aging in any organism represents an exciting opportunity in aging research. Limited studies on lncRNAs in aging in *Drosophila*, and the emerging importance of circRNAs may suggest the need for identifying new noncoding RNAs and mechanisms in aging. The possible involvement of various RNA modifications, such as m^6^A and m^5^C, as well as RNA editing in aging remains elusive and warrant further investigation.

## CONFLICT OF INTEREST

The authors declare that they have no conflict of interest.

## AUTHOR CONTRIBUTIONS

**Amy Tsurumi:** Supervision; writing‐original draft; writing‐review and editing. **Willis Li:** Supervision; writing‐original draft; writing‐review and editing.

### PEER REVIEW

The peer review history for this article is available at https://publons.com/publon/10.1002/ggn2.10026.

## Supporting information

Transparent‐Peer‐Review‐Record
