# Erratum

**DOI:** 10.1002/ggn2.10032

**Published:** 2020-09-07

Tsurumi, A, Li, WX. Aging mechanisms—A perspective mostly from *Drosophila*. *Advanced Genetics*. 2020; 1:e10026. https://doi.org/10.1002/ggn2.10026

In the originally published version of this article, Shriners Hospitals Research Funding was misattributed to Willis Li in the Acknowledgments section. The correct funding recipient is Amy Tsurumi. The correct acknowledgments appear below. The publisher regrets the error.

