# Supplementary material for: Aging mechanisms—A perspective mostly from Drosophila
Source: Adv Genet (Hoboken). 2020 Aug 10;1(1):e10026. doi: 10.1002/ggn2.10026 (PMC9744567; doi:10.1002/ggn2.10026)
Supplement: Supplementary file 1 — Transparent‐Peer‐Review‐Record [file GGN2-1-e10026-s001.pdf]

Advanced Genetics

Epigenetic alterations in aging, a mostly *Drosophila* perspective

Amy Tsurumi * and Willis X. Li

*Corresponding author

Review timeline

Submission date: 09/16/2019

1st Editorial decision: Minor Revision – 11/14/2019
1st Revision received: 01/23/2020
2rd Editorial decision: Minor Revision – 02/20/2020
2rd Revision received: 03/04/2020
3rd Editorial decision: Minor Revision – 03/26/2020
3rd Revision received: 04/03/2020
Accepted: 04/08/2020

Editor: Alison Liu

**1st Editorial decision**	**11/14/2019**

Editor's recommendations:

This Perspective represents fairly the main concepts and advances in the field of the aging - related epigenetics in human and Drosophila. It clarifies several important concepts in the aging biology and summarizes the major findings, particularly in Drosophila, and points out the gaps and future approaches. The authors also indicate the technical difficulties in the human aging studies and the advantages using Drosophila to advance the field. We think overall this Perspective is valuable to the aging community and would like to publish it with some revisions. Based on reviewers' comments, we suggest reorganizing the article in the manner that is more logic and concise, consolidate related information under proper subheadings, and add a couple of tables and columns to strengthen the arguments. The goal is to inspire researchers to adopt Drosophila in their research or make use of the knowledge yielded from this model organism. As suggested by the reviewers, some rigorous proof readings are also needed to remove grammar mistakes and add back the missing references. Please see below for the detail.

Section 1. Please also explain the difference and overlap in terms of processes and mechanisms between "senescence" and "aging", as used in the aging field. Please also consider including a simple table indicating the markers (or measurements) used to measure the healthspan in Drosophila, and humans, or both, including epigenetic markers. You need to indicate the common mechanisms to stress that Drosophila is an attractive model to study healthspan (doi: 10.14336/AD.2018.1030). As suggested by the reviewer, you need to be cautious about "precise life-style recommendations". Please also improve

the clarity of Figure 3. As suggested by the reviewer, it is better to discuss aging vs aging diseases after "Lifespan" and "healthspan", and how diseases can change both.

Section 2. Change "3b" into a new Section 2 to emphasize how Drosophila has contributed mechanistic insights into aging processes due to its genetic conservation with mammals, and its ability to react to common environmental factors as humans with better resolution. You may use a simple table to make your point by listing major genes or pathways, pharmacological interventions, and environmental factors, as suggested by the reviewer.

Section 3. Combine the Section 2 and 3 (apart from previous 3b) as the new Section 3 to discuss the epigenetic aspects of the aging-related epigenetics in human and Drosophia side by side. Here is the order I recommend:

a. Combine the discussions in the first paragraph of "2a", "2b", "2c", and "3c" on heterochromatin alterations and regulations. Add 2-3 sentences explaining the term, "heterochromatin", in contrast to "euchromatin". This ensures the discussion of heterochromatin and histone modification before DNA methylation, emphasizing its universal aspects and important roles in Drosophila; there is very little DNA methylation (as in nematode and budding Yeast). Since you provide a lot of information, it will be helpful for readers if you organize topics under different subheadings, for example:
    (i) Compare and contrast heterochromatin loss and epigenetic landscape changes in Drosophila and Humans and what that tells us of the mechanisms operating in aging cells.
    (ii) Narrate the regulatory mechanisms of age-related heterochromatin loss in separate paragraphs.
    ---The tumor suppressor p16. P16 plays an important role in cell cycle and senescence and aging process. A few studies have shown that p16 can regulate heterochromatin distribution. Please briefly summarize these findings and add key references.
    ---Non-coding RNA. Non-coding RNAs play a pivotal role in regulating 3D chromatin structure, enhancer-promoter interactions, and gene expression. How do non-coding RNA alterations associate with the heterochromatin loss?
    ---RNAi pathways and small non-coding RNAs as indicated.
    ---Lamin B. You might want to add a category about Nuclear Lamina in the Table because Lamin B has been shown recently to play an important role in regulating heterochromatin in Drosophila and mammals, doi: 10.1111/acel.12465; doi: 10.1016/j.ceb.2016.03.004; doi: 10.1186/s13072-018-0235-8). Briefly indicate its normal roles and as a regulator of heterochromatin.
    (iii) Include a small table on the drugs regulating heterochromatin and Sirturin pathways in Drosophila and humans.

b. Please reorganize the alterations of histone modifications and their regulations during the aging process in Drosophila and mammals into different paragraphs (or under different subheadings, see below). Please add one column in the Table to show the conserved human epigenetic regulators and common aging phenotypes, which can help stress the importance of Drosophila. Please try to discuss antagonistic pleiotropy of heterochromatin loss and epigenetic alternations aging.
    (i) H3k27me3, the repressive mark
    (ii) H3k9me3, H3k4me3, and H3k36me3, the activation marks and their interactions with H3k27me3
    (iii) The Sirturin family of histone deacetylases and their regulators including drugs. Sirtuins are important aging genes with the linkage to stress pathways, NAD, TE activation pathways, as

indicated in the text. It would be nice to briefly indicate the conservation between human and Drosophila in this gene family.

c. MiRNAs involved in regulating the aging process through targeting the developmental pathways. Any other miRNAs besides miR-34?
d. Combine the discussions in “2a”, “3d”, and “2d” about the DNA methylation changes during the aging process and aging clocks. You might consider to re-draft “2a” to discuss, instead, whether DNA methylation plays a causal role or simply a consequence during the aging process in mammals; a recent paper showed that histone methyltransferase had a positive role in setting the aging ricking rate by changing DNA methylation (https://doi.org/10.1186/s13059-019-1753-9).

This is the place to introduce and discuss the connections and the roles of other chemical modifications in DNA and RNA, including m6A (doi: 10.1530/JME-19-0021) in the aging process and whether these modification function as the potential substitutes in Drosophila for the m5C. What is the potential to measure these in a range of organisms as an aging clock? Please also put these modifications under a right category in the Table.

Section 4. Change “3a” into “4b” and move it under the Section 4, in which you discussed general considerations for future studies of aging. Please add citations if the recommended approaches have been published recently. It is a good place to discuss the advantages of the Drosophila system as demonstrated in “3a”. The Perspective needs to end with your recommendations for new directions and experimental approaches using the Drosophila system. Where relevant to mammalian and human aging studies, please point out where they are complementary or mutually supporting.

**1st review**	**01/07/2020**

Reviewer(s)' Comments to Author:

Reviewer: 1

Tsurumi and Li Kong reviewed epigenetic alterations in aging, a mostly *Drosophila* perspective. It will enhance our knowledge of epigenetics in aging. This manuscript will be of general interest to the readers of ggn journal. But it seems to me some contents are not logical and need to be re-organized. In addition, I have some concerns need to be addressed.

My comments:

1. Page4, Life expectancy has more than doubled overall in the past century due to improved measures for infectious diseases control and enhanced quality of life (ref). Please add reference.
2. Page 6: you mentioned that there are various different particularly measurements of healthspan. It is better to list relevant indicators that should be tested for healthspan in animal models, especially in *Drosophila*.
3. Page 8: the term “hemi-methylation”, please define what does mean.
4. Page 8, Add references after “Further investigation on the effect of …….steroid receptors were found to be associated with significantly accelerated DNAm age.”
5. In this part, 1c. “Lifespan” and “healthspan” as distinct aging phenotypes”, mainly

described the two distinct aging phenotypes “Lifespan” and “healthspan”. But paragraph 2 “Approaches for targeting aging to prevent aging-related diseases would first require…..” was focus on the relationship between aging and agingrelated diseases. Thus, I suggest that this section could move to “1b. Recent considerations of aging versus aging-related diseases.”

6. In 2a section, in my opinion, some of contents were redundant. Please rearrange this section

7. P11, as a tumor suppressor, P16 protein is crucial for cancer protection, and induces cell cycle arrest and senescence, and shown to be a major contributor to aging [25-27]. Please clarify clearly what is the relationship between the P16 protein and heterochromatin redistribution in the aging process.

8. P18: you`re talking about RNAi pathway components Dicer-2 and Ago2, then described Dnmt2, the moved back to Ago2. It was not logical. I think that Dnmt2 can be talked after you finish describing of the Dicer-2 and Ago2.

9. In 3a, please merge the 1st and the 2nd paragraph into one paragraph and reduce content.

10. In 3b, please describe what are the conserved genetic factors in multiple species.

11. In part 3c, it should be more subtitles, it will make readers easy to follow. This part was too long, especially the first and the second paragraph. Please reduce text according to Table 1. If the contents had already appeared in the table 1, it was not necessary to elaborate too much in manuscript.

12. P23, “On the other hand, because Drosophila appears to lack 5mC during adulthood, it may provide a means for studying other epigenetic marks without the concerns of effects due to cross-talk with DNA methylation.”

I believe it should be “very low level”, rather than lack and please add the references, such as

Capuano, F.; Mulleder, M.; Kok, R.; Blom, H. J.; Ralser, M., Cytosine DNA methylation is found in Drosophila melanogaster but absent in Saccharomyces cerevisiae, Schizosaccharomyces pombe, and other yeast species. *Anal Chem* **2014,** 86, (8), 3697-702.

Lian, T.; Gaur, U.; Wu, Q. I.; Tu, J.; Sun, B.; Yang, D.; Fan, X.; Mao, X.; Yang, M., DNA methylation is not involved in dietary restriction induced lifespan extension in adult Drosophila. *Genetics research* **2018,** 100, e1.

13. Please revise “18oC, 25oC and 29oC” to “18℃, 25℃ and 29℃, respectively.

14. All the species and genus names should be modified as italic in full text, such as Drosophila melanogaster, Drosophila, and C.elegans.

Reviewer: 2

Comments to the Author

“Epigenetic alternations in aging, a mostly Drosophila perspective” is an expansive and wide ranging review of the current understanding of how epigenetic mechanisms may influence aging. The review does a nice job of providing an introduction to the different considerations for the study of aging before delving into a pretty detailed review of studies in Drosophila, and using these to motivate suggestions for further studies. I found the review informative, but unfortunately, very difficult to read due to numerous errors that can (and should) be relatively easily fixed by proper copy editing and some more rigorous and clear organization. There are many questionable grammar choices and the fact that

multiple references still simply have "ref" written on them suggest that the authors haven't actually submitted a final draft. Another example of careless writing that appears in section 2 is the use of 6mA and m6A to refer to the same methylated base. The review has good information, and if the authors can clean up the language and edit it for clarity/readability, it should be a great summary of the field.

The authors start by motivating the study of aging while briefly reviewing common terms in the field. I found this section ("Towards increasingly rigorous definitions of terms in aging research") the most compelling of the review, as it not only clearly provides strong justifications for the study of aging, but does a really nice job of introducing the field as well. The section also does well to provide clarification on the terminology that will follow in the review, and clearly explains the need for distinguishing some nebulous ideas like aging vs. aging-related diseases, and healthspans and lifespans. I also appreciated how the authors cleverly applied the "causal pie" model to help justify the study of aging as a promising means for the treatment of multiple causes of morbidity and mortality.

In the following section, the authors review the convincing evidence that spotlights epigenetics as an important line of inquiry in further understanding the processes that are involved in aging. The authors carefully cover direct modifications to DNA itself before delving into different histone modifications that have been shown to be relevant to aging. Throughout there is commentary on the role of tissue specificity in aging and the role that tissue specific mechanisms might play in the aging process. Overall, the authors do well to highlight the role of DNA methylation, chromatin/histone regulation, and transposon activation/repression as major themes for the field. I did find it a bit strange that mechanisms that involve non-coding RNA was singled out as a separate mechanism instead of being integrated into the other sections as being another way in which DNA methylation, chromatin/histone regulation, and transposon activity are controlled, but this is well within reason and up to the author's discretion. Additionally, in section 2b, there is significant exposition of senescence vs. aging in which antagonistic pleiotropy is not brought up, which also seems rather inefficient.

The following section is a deep dive into the role that research using Drosophila has played in the field. As someone who uses Drosophila in their research, I found section 3a compelling and well written, but I found that the following sections lacked sufficient organization to be very helpful. The level of detail that the authors dive into is difficult to keep up with and the writing does little to mitigate this. For example, section 3c starts with a paragraph that is two pages long and seems to meander among multiple different topics. There is clearly great information to be presented, but the writing and organization hampers rather than helps the delivery. In terms of specific decisions the authors made, I would have appreciated a fuller discussion of the role of sirtuin-activating compounds in fly research, as that topic had much more controversy than suggested by the authors (e.g. as was one of the major conclusions of their reference #94), and while I generally agree with the assertions the authors make about the utility of Drosophila model research in human applications, I'd suggest more caution, particularly for "precise lifestyle recommendations".

The authors proceed to a more general discussion of what areas of study might provide the best insights

into the topics they covered in the previous two sections. These are well motivated, and it's difficult to argue against their importance as the authors explain.

Reviewer: 3

Comments to the Author
This review focuses on the epigenetic mechanisms of aging, with a particular focus on the Drosophila model. The review is divided into four sections: 1) an overview of approaches and definitions in aging research, 2) a discussion of many epigenetic mechanisms related to aging in general, 3) a discussion of the strengths of the Drosophila model as well as extensive literature review of epigenetic-related results in this organism, and 4) a summary/perspective/future work section. The review is well-organized, and especially section 3 summarizing the Drosophila epigenetic aging literature is very valuable. I have some minor suggestions and comments.

1. Overall the manuscript would benefit from a careful proof reading. There are numerous grammatical mistakes present which sometimes impinge on comprehension. To cite only one specific example, the last paragraph on page 9 (starting "For elucidating loci") was very hard to parse, and the first sentence doesn't make sense as written. The paper would be much stronger if these distractions were removed to promote easier reading. Also note there is a missing reference (with "(ref)" placeholder) near the end of page 6.

2. Figure 3 is not very clear, and I question whether it is really necessary? I can get the gist of it by reading the text, but the figure does not stand alone as currently constituted. I would suggest removing it, or at the least fleshing out the figure legend so it's more clear what it's actually trying to say. To me at least, it seems to be needlessly complicating the general idea.

3. Section 2c is a little weak and hand-wavy. There are a few studies cited, but the results aren't really described well. I think adding a few sentences that the actual results would greatly help this section.

4. One potential epigenetic mechanism that is not discussed is RNA editing. I would consider adding a short section about this mechanism as it may prove interesting and has been linked to lifespan in at least one fly paper (Savva et al 2013 Nat Comm). Section 2d would be a logical place together with the current discussion of RNA methylation.

5. Section 3b makes an important point but it's mostly glossed over. There aren't any citations in this section and it's a little vague. Adding a few concrete examples to link flies and mammals would be helpful. Off the top of my head, this could be done pretty easy with both dietary (calorie restriction etc.) as well as pharmacological (resveratrol, rapamycin, etc) interventions.
6. In 4b it is worth remarking that single cell approaches are useful not only for methylation studies, but will most likely be crucial for epigenetic and transcriptomic studies as well. The field is moving away from bulk work and trying to use new technologies to parse apart the cell type-specific effects of aging.

Overall this is a good review and makes a strong case for, and does a good job describing, the strengths of the Drosophila model system for aging research in general and epigenetic approaches specifically.

1st Author Response to Reviewers and Editor	01/28/2020

Author Point-by-point Response to Reviewers

Reviewer #1:

"1. Page 4, Life expectancy has more than doubled overall in the past century due to improved measures for infectious diseases control and enhanced quality of life (ref). Please add reference."
Author: We apologize for having omitted the reference number and have provided it in the revised draft.

"2. Page 6: you mentioned that there are various different particularly measurements of healthspan. It is better to list relevant indicators that should be tested for healthspan in animal models, especially in Drosophila."
Author: We provided a list of relevant healthspan indicators, as suggested in the section, as well as provided a new Table 1 listing measures of healthspan and relating them to humans.

"3. Page 8: the term "hemi-methylation", please define what does mean."
Author: We provided a definition for this term and added a reference. We hope this is helpful, thank you for the suggestion.

"4. Page 8, Add references after "Further investigation on the effect of …….steroid receptors were found to be associated with significantly accelerated DNAm age.""
Author: We added the reference number for this finding. Thank you for pointing out this omission.

*"5. In this part, 1c. "Lifespan" and "healthspan" as distinct aging phenotypes", mainly described the two distinct aging phenotypes "Lifespan" and "healthspan". But paragraph 2 "Approaches for targeting aging to prevent aging-related diseases would first require….." was focus on the relationship between aging and aging-related
diseases. Thus, I suggest that this section could move to "1b. Recent considerations of aging versus aging-related diseases."*
Author: We are grateful to Reviewer #1 for this suggestion and moved the section accordingly, and restructured the two sections.

"6. In 2a section, in my opinion, some of contents were redundant. Please rearrange this section."
Author: We rearranged this section and hope it is less redundant.

"7. P11, as a tumor suppressor, P16 protein is crucial for cancer protection, and induces cell cycle arrest and senescence, and shown to be a major contributor to aging [25-27]. Please clarify clearly what is the relationship between the P16 protein and heterochromatin redistribution in the aging process."
Author: We thank Reviewer #1 for bringing up this point and provided further clarification of the relationship between p16 protein and SAFH. Furthermore, we restructured the sections and added more detail.

"8. P18: you`re talking about RNAi pathway components Dicer-2 and Ago2, then described Dnmt2, the moved back to Ago2. It was not logical. I think that Dnmt2 can be talked after you finish describing of the Dicer-2 and Ago2."
Author: We are grateful to Reviewer #1 for this suggestion and re-organized the section accordingly. We also added headings and segmented the section and hope these modifications help with clarity.

"9. In 3a, please merge the 1st and the 2nd paragraph into one paragraph and reduce content."
Author: We combined the first and second paragraphs of section 3a and reduced the content, as suggested. Furthermore, we moved this section.

"10. In 3b, please describe what are the conserved genetic factors in multiple species."
Author: We mentioned that these include sirtuins and other chromatin regulators, insulin signaling and mTOR pathway components, as suggested. In section 1c.ii. and section 3a, we elaborate on some of these factors.

"11. In part 3c, it should be more subtitles, it will make readers easy to follow. This part was too long, especially the first and the second paragraph. Please reduce text according to Table 1. If the contents had already appeared in the table 1, it was not necessary to elaborate too much in manuscript."
Author: We added more sections and segmented the first and second paragraphs, as well as reduced the text to summarize the information provided in Table 1.

"12. P23" … "I believe it should be "very low level", rather than lack and please add the references, such as…"
Author: We changed the description of DNA methylation in flies from "lack of" to "very low level" as suggested and added the two references – we are grateful to Reviewer #1 for this suggestion.

"13. Please revise "18oC, 25oC and 29oC" to "18℃, 25℃ and 29℃, respectively."
Author: We thank Reviewer #1 for pointing this out and have made the suggested edits.

"14. All the species and genus names should be modified as italic in full text, such as Drosophila melanogaster, Drosophila, and C.elegans."
Author: We italicized the species and genus names – we apologize that they were not earlier.

Reviewer #2:

"I found the review informative, but unfortunately, very difficult to read due to numerous errors that can (and should) be relatively easily fixed by proper copy editing and some more rigorous and clear organization…."

Author: We are grateful to Reviewer #2 for acknowledging that our review is informative and apologize for the grammatical errors. We spent significant effort to edit our draft and hope it is much easier to read and is free of errors.

"Another example of careless writing that appears in section 2 is the use of 6mA and m6A to refer to the same methylated base."

Author: We used "6mA" to refer to DNA methylation and "m6A" to refer to RNA methylation – although they are modifications of the same base, conventionally, they are differentiated by these abbreviations. In order to clarify this, we added a reference (Li et al., *International J. of Mol. Sci.,* 2019) and added a sentence to explain these differences in part 2e's second paragraph.

"… The review has good information, and if the authors can clean up the language and edit it for clarity/readability, it should be a great summary of the field."

Author: We thank Reviewer #2 for the encouragement and hope that the revised version is more clear.

"The authors start by motivating the study of aging while briefly reviewing common terms in the field. I found this section ("Towards increasingly rigorous definitions of terms in aging research") the most compelling of the review, as it not only clearly provides strong justifications for the study of aging, but does a really nice job of introducing the field as well. The section also does well to provide clarification on the terminology that will follow in the review, and clearly explains the need for distinguishing some nebulous ideas like aging vs. aging-related diseases, and healthspans and lifespans. I also appreciated how the authors cleverly applied the "causal pie" model to help justify the study of aging as a promising means for the treatment of multiple causes of morbidity and mortality.".

Author: We are grateful to Reviewer #2 for recognizing the importance of the information in our paper.

In the following section, the authors review the convincing evidence that spotlights epigenetics as an important line of inquiry in further understanding the processes that are involved in aging. … Overall, the authors do well to highlight the role of DNA methylation, chromatin/histone regulation, and transposon activation/repression as major themes for the field. I did find it a bit strange that mechanisms that involve non-coding RNA was singled out as a separate mechanism instead of being integrated into the other sections as being another way in which DNA methylation, chromatin/histone regulation, and transposon activity are controlled, but this is well within reason and up to the author's discretion.

Author: We thank Reviewer #2 for the suggestions for organizing our review. Indeed, as Reviewer #2 mentions, non-coding RNA could be described as being among loci that are controlled by DNA methylation and chromatin/histone regulation. Therefore, we mentioned this point of how non-coding RNA alterations relate to chromatin regulation as well as provided additional information in section 2d.

Additionally, in section 2b, there is significant exposition of senescence vs. aging in which antagonistic pleiotropy is not brought up, which also seems rather inefficient."
Author: We thank Reviewer #2 for this suggestion and added the concept of antagonistic pleiotropy in section 2c.

"The following section is a deep dive into the role that research using Drosophila has played in the field. As someone who uses Drosophila in their research, I found section 3a compelling and well written, but I found that the following sections lacked sufficient organization to be very helpful. The level of detail that the authors dive into is difficult to keep up with and the writing does little to mitigate this. For example, section 3c starts with a paragraph that is two pages long and seems to meander among multiple different topics. There is clearly great information to be presented, but the writing and organization hampers rather than helps the delivery."
Author: Again, we thank Reviewer #2 for the positive comment and hope that the re-organization and editing is helpful for part 3, as well as in general.

"In terms of specific decisions the authors made, I would have appreciated a fuller discussion of the role of sirtuin-activating compounds in fly research, as that topic had much more controversy than suggested by the authors (e.g. as was one of the major conclusions of their reference #94), and while I generally agree with the assertions the authors make about the utility of Drosophila model research in human applications, I'd suggest more caution, particularly for "precise lifestyle recommendations"."
Author: We expanded part 3a to include a section about sirtuin-activating compounds. We also agree that the wording of "precise lifestyle recommendations" is not appropriate and modified the wording.

Reviewer #3:

"… The review is well-organized, and especially section 3 summarizing the Drosophila epigenetic aging literature is very valuable. I have some minor suggestions and comments."
Author: We thank Reviewer #3 for recognizing the value of our work and for spending the time to provide constructive comments.

1. Overall the manuscript would benefit from a careful proof reading. There are numerous grammatical mistakes present which sometimes impinge on comprehension. To cite only one specific example, the last paragraph on page 9 (starting "For elucidating loci") was very hard to parse, and the first sentence doesn't make sense as written. The paper would be much stronger if these distractions were removed to promote easier reading. Also note there is a missing reference (with "(ref)" placeholder) near the end of page 6.
Author: We thank Reviewer #3 for recognizing the value of our work and for spending the time to provide constructive comments. We apologize for the omission of the reference and the grammatical errors, and hope that this revised version is improved.

2. Figure 3 is not very clear, and I question whether it is really necessary? I can get the gist of it by reading the text, but the figure does not stand alone as currently constituted. I would suggest removing it, or at the least fleshing out the figure legend so it's more clear what it's actually trying to say. To me at least, it seems to be needlessly complicating the general idea.
Author: We are grateful to Reviewer #3 for bringing this point up and provided further information in the figure and the figure legend (now Figure 2A), as well as restructured part 1 to elaborate on the relevance of the application of model in part 1c.ii.

3. Section 2c is a little weak and hand-wavy. There are a few studies cited, but the results aren't really described well. I think adding a few sentences that the actual results would greatly help this section.
Author: We moved the section to 2d and added more information describing the relevance of non-coding RNAs, as well as provided Table with additional information. We hope that these modifications help improve this work.

4. One potential epigenetic mechanism that is not discussed is RNA editing. I would consider adding a short section about this mechanism as it may prove interesting and has been linked to lifespan in at least one fly paper (Savva et al 2013 Nat Comm). Section 2d would be a logical place together with the current discussion of RNA methylation.
Author: We are grateful to Reviewer #3 for this suggestion and included a section on RNA editing and added/elaborated on the suggested citation.

5. Section 3b makes an important point but it's mostly glossed over. There aren't any citations in this section and it's a little vague. Adding a few concrete examples to link flies and mammals would be helpful. Off the top of my head, this could be done pretty easy with both dietary (calorie restriction etc.) as well as pharmacological (resveratrol, rapamycin, etc) interventions.
Author: We elaborated on this section and provided the concrete examples that Reviewer #3 suggested. We thank Reviewer #3 for aiding in improving our work.

6. In 4b it is worth remarking that single cell approaches are useful not only for methylation studies, but will most likely be crucial for epigenetic and transcriptomic studies as well. The field is moving away from bulk work and trying to use new technologies to parse apart the cell type-specific effects of aging.
Author: We included this point in 4b and thank Reviewer #3 for this idea.

"Overall this is a good review and makes a strong case for, and does a good job describing, the strengths of the Drosophila model system for aging research in general and epigenetic approaches specifically."
Author: We are grateful to Reviewer #3 for the positive words and hope the revised version addresses the suggested points.

Point-by-point Response to the Editors' recommendations

"This Perspective represents fairly the main concepts and advances in the field of the aging - related epigenetics in human and Drosophila. It clarifies several important concepts in the aging biology and summarizes the major findings, particularly in Drosophila, and points out the gaps and future approaches. The authors also indicate the technical difficulties in the human aging studies and the advantages using Drosophila to advance the field. We think overall this Perspective is valuable to the aging community and would like to publish it with some revisions. …"
Author: We are grateful for this opportunity and thank the Editors for the suggestions to improve our work, as well as the Reviewers for their input.

"Section 1. Please also explain the difference and overlap in terms of processes and mechanisms between "senescence" and "aging", as used in the aging field. Please also consider including a simple table indicating the markers (or measurements) used to measure the healthspan in Drosophila, and humans, or both, including epigenetic markers. You need to indicate the common mechanisms to stress that Drosophila is an attractive model to study healthspan (doi: 10.14336/AD.2018.1030). As suggested by the reviewer, you need to be cautious about "precise life-style recommendations". Please also improve the clarity of Figure 3. As suggested by the reviewer, it is better to discuss aging vs aging diseases after "Lifespan" and "healthspan", and how diseases can change both."
Author: We are thankful to the Editors for their advice. We have added Table 1 to indicate healthspan measures in *Drosophila* as they may relate to human measures. We have also added information about the relevance of the epigenetic marks we highlight in *Drosophila* studies in Tables 2-4. Also, we modified the wording of "precise life-style recommendations."

"Section 2. Change "3b" into a new Section 2 to emphasize how Drosophila has contributed mechanistic insights into aging processes due to its genetic conservation with mammals, and its ability to react to common environmental factors as humans with better resolution. You may use a simple table to make your point by listing major genes or pathways, pharmacological interventions, and environmental factors, as suggested by the reviewer."
Author: Since we extensively rearranged various sections, we included the discussion about environment-genetic interactions that are conserved in Section 3a. We provided a better explanation of how the use of *Drosophila* may help in assessing environmental factors and interactions with genetic pathways with better resolution.

"Section 3. Combine the Section 2 and 3 (apart from previous 3b) as the new Section 3 to discuss the epigenetic aspects of the aging-related epigenetics in human and Drosophia side by side….
Combine the discussions in the first paragraph of "2a", "2b", "2c", and "3c" on heterochromatin alterations and regulations. Add 2-3 sentences explaining the term, "heterochromatin", in contrast to "euchromatin". This ensures the discussion of heterochromatin and histone modification before DNA methylation, emphasizing its universal aspects and important roles in Drosophila; there is very little DNA methylation (as in nematode and budding Yeast). Since you provide a lot of information, it will be helpful for readers if you organize topics under different subheadings… for example…"
Author: We are grateful to the Editors for their suggestions and have extensively rearranged the section, as well as added sub-headings, which we hope would improve our organization. Furthermore, we

added more information about hetero- vs. euchromatin. We are grateful to the Editors for their suggestions in adding further information and references about: the role of P16 in senescence; lncRNA and heterochromatin; relationship between nuclear lamins. We have included these information and hope that these additions will improve our manuscript.

"Include a small table on the drugs regulating heterochromatin and Sirturin pathways in Drosophila and humans."
Author: We have not added this table, but rather explained the interaction between these drugs (and other environmental factors) and genetic interactions in section 3a, as we believed it would provide more information.

"Please reorganize the alterations of histone modifications and their regulations during the aging process in Drosophila and mammals into different paragraphs (or under different subheadings, see below). Please add one column in the Table to show the conserved human epigenetic regulators and common aging phenotypes, which can help stress the importance of Drosophila..."
Author: We added subsections to our discussion about chromatin regulation in *Drosophila* during aging, as well as added information about human studies in our summary Tables 2-4.

"MiRNAs involved in regulating the aging process through targeting the developmental pathways. Any other miRNAs besides miR-34?"
Author: We added information about additional miRNAs.

Combine the discussions in "2a", "3d", and "2d" about the DNA methylation changes during the aging process and aging clocks. You might consider to re-draft "2a" to discuss, instead, whether DNA methylation plays a causal role or simply a consequence during the aging process in mammals; a recent paper showed that histone methyltransferase had a positive role in setting the aging ricking rate by changing DNA methylation (https://doi.org/10.1186/s13059-019-1753-9).
This is the place to introduce and discuss the connections and the roles of other chemical modifications in DNA and RNA, including m6A (doi: 10.1530/JME-19-0021) in the aging process and whether these modification function as the potential substitutes in Drosophila for the m5C."
Author: We have rearranged these sections accordingly and added information about the reference suggested.

"What is the potential to measure these in a range of organisms as an aging clock?"
Author: We have omitted answering this, as we were unable to provide a solid argument.

Section 4. Change "3a" into "4b" and move it under the Section 4, in which you discussed general considerations for future studies of aging. Please add citations if the recommended approaches have been published recently. It is a good place to discuss the advantages of the Drosophila system as demonstrated in "3a". The Perspective needs to end with your recommendations for new directions and experimental approaches using the Drosophila system. Where relevant to mammalian and human aging studies, please point out where they are complementary or mutually supporting.

Author: We added a last section with recommended future studies using flies. We have also added references and hope they are sufficiently complete.

2nd Editor's decision – revise with minor revisions	February 20, 2020

Editor Comments to Author:

Thank you for revising the manuscript with additional information, references, discussions, and new ideas. This serves our goal to inspire researchers to use or make use of the knowledge yielded from this great model organism for understanding aging and aging-related diseases and finding interventions. To further improve it, we have done extensive editing and marked all the changes and suggestions in details in the text and tables (see the attached files), intending to reduce you time and efforts for revision. Here I summarize a few major points for making these changes.

(1) The Perspective should have a strong focus on using Drosophila to tackle the aging problems related to humans, so the discussions related to the aging mechanisms that are not conserved with Drosophila should be reduced to the minimum. This applies to the 5mC DNA methylation aging clock discussion that is mostly unrelated to Drosophila (see the edits in the text). This is the same for the human p16 discussion.

(2) It is better to put the sections about DNA and RNA chemical modifications after the discussion about heterochromatin because very little is known about their association with aging. However, we will leave this for you to decision as it is not crucial.

(3) Please combine the section "Use of the Drosophila as a model organism for aging research" with the section, "Established assays and methodologies for aging in Drosophila and relating them to the proposed human Healthspan index". Both sections are related and about the advantage of using Drosophila to understand the aging problem.

(4) Please remove Figure 2A because this is a redraw of the figure published previously. We prefer a version with the inputs of new ideas if you would replace it.

(5) Please double-check all the writings and references for their accuracy. I suggest that you discuss with Dr. Willis Li during the revision, particularly about the contents related to JNK and STATs and Drosophila genetics and ask him to help with polishing the text.

If you have any question regarding to our edits and any other questions, please do not hesitate to contact me.

2nd Author Response to Editor	March 4, 2020

Point-by-point Response to the Editors

"This serves our goal to inspire researchers to use or make use of the knowledge yielded from this great model organism for understanding aging and aging-related diseases and finding interventions. To further improve it, we have done extensive editing and marked all the changes and suggestions in details in the text and tables (see the attached files), intending to reduce you time and efforts for revision."

Author: We are extremely grateful to you for the opportunity to improve our work and for all your assistance and guidance in editing our draft. We hope we have sufficiently addressed the helpful points that you have pointed out throughout our text and tables.

1) The Perspective should have a strong focus on using Drosophila to tackle the aging problems related to humans, so the discussions related to the aging mechanisms that are not conserved with Drosophila should be reduced to the minimum. This applies to the 5mC DNA methylation aging clock discussion that is mostly unrelated to Drosophila (see the edits in the text). This is the same for the human p16 discussion."

Author: We thank you for this suggestion – we have cut down significantly on explaining mechanisms that are not conserved in Drosophila; and have moved the sections about 5mC DNA methylation and human p16-mediated senescence, where it is now discussed as a comparison to the Drosophila model, rather than a standalone explanation. We hope that these restructuring and changes help focus this work, thank you so much for this suggestion.

(2) It is better to put the sections about DNA and RNA chemical modifications after the discussion about heterochromatin because very little is known about their association with aging. However, we will leave this for you to decision as it is not crucial.

Author: We are grateful for this suggestion and have moved the discussion about heterochromatin and histone modifications to come before DNA/RNA modifications.

(3) Please combine the section "Use of the Drosophila as a model organism for aging research" with the section, "Established assays and methodologies for aging in Drosophila and relating them to the proposed human Healthspan index". Both sections are related and about the advantage of using Drosophila to understand the aging problem.

Author: We have restructured our perspective significantly, and have combined sections relevant to using Drosophila to understand human aging, while taking out redundant sections and those that are only relevant to humans.

(4) Please remove Figure 2A because this is a redraw of the figure published previously. We prefer a version with the inputs of new ideas if you would replace it.

Author: We have kept the figure and have explained that the figure represents a new application of an established modeling method to the field of aging. We believe that the idea presented is novel, as there are no precedence of suggestions of applying the causal pie model to understand aging and aging-related diseases; and we believe it will help bridge public health and basic science. We apologize that this was not clearly explained before and hope that the edits help highlight the new aspect of this figure.

(5) Please double-check all the writings and references for their accuracy. I suggest that you discuss with Dr. Willis Li during the revision, particularly about the contents related to JNK and STATs and Drosophila genetics and ask him to help with polishing the text.

Author: Thank you very much for your suggestions – we apologize for the errors and hope that this revised version is significantly improved. We are very grateful to you for taking the time to provide a lot of constructive input.

**3rd editor decision - accept in principle with minor revisions**	**March 26, 2020**

Editor Comments to Author:

Thank you for making efforts to revise the paper. I am happy to accept-in-principle your manuscript. However, before we can send the paper to our production team, we still need a little more work to polish the text. But you are almost there! These edits intend to make the discussions more concise and remind you of minor issues that still need to be clarified - I suggest that you avoid using long sentences with complicated structure that sometimes interrupt the flow. Please look at the edits in the text and tables and make change accordingly, if acceptable. At this stage, you have made all your points, so additional writings and citations are not necessary.

Please follow the Guide to Authors on Formatting to include the citation numbers in the text, which will appear in print like this:

"These pathways include sirtuins and other chromatin regulators (Table 2), inflammation24, oxidative stress detoxification25, insulin signaling26 and mammalian Target of Rapamycin (mTOR) pathway27. "

In addition, Ref 7 is an editorial and has no author:

7. Anon. 2016 Alzheimer's disease facts and figures. Alzheimers Dement. 2016;12(4):459-509. doi: 10.1016/j.jalz.2016.03.001. PubMed PMID: 27570871.

The edited Tables have the R2 reference numbers corrected and need to be checked.

Finally, please consider to modify the title to reflect the content that covers a large portion unrelated to epigenetics.

Please feel free to contact me if you have any question.

3rd author response to editor	April 3rd, 2020

Point-by-point Response to the Editor

We are extremely grateful to you for all your comments, editing and guidance. We hope we have sufficiently addressed the areas requiring clarification and improvements. We have accepted your edits and suggestions. Areas that we have additionally worked on are highlighted in yellow. We have addressed the points that you have commented on in our document and tables throughout. Thank you very much for the opportunity to further improve our work.

"Please follow the Guide to Authors on Formatting to include the citation numbers in the text …"
Author: Thank you for pointing this out – we have referred to the Guide and changed the citations to superscript numbers and Vancouver reference list.

"… Ref 7 is an editorial and has no author…"
Author: We apologize that we did not reference this article correctly – thank you for letting us know how to correctly reference it.

"The edited Tables have the R2 reference numbers corrected and need to be checked"

Author: We apologize that we had not provided the correct numbers – we re-did the Endnote referencing for all the text and tables.

"Finally, please consider to modify the title to reflect the content that covers a large portion unrelated to epigenetics."

Author: We have provided an alternative title. However, we have additional suggestions below, for which we would be grateful for your input.

"Aging, epigenetic alterations, and model organisms"

"Aging, epigenetic alterations, and the *Drosophila* model"

"Molecular mechanisms of aging – a prospective mostly from *Drosophila*"

"Aging – a prospective mostly from *Drosophila*"
